# Directionality of neural activity in and out of the seizure onset zone in focal epilepsy

**DOI:** 10.1162/netn_a_00454

**Published:** 2025-06-30

**Authors:** Hamid Karimi-Rouzbahani, Aileen McGonigal

**Affiliations:** Neurosciences Centre, Mater Hospital, South Brisbane, Australia; Mater Research Institute, University of Queensland, South Brisbane, Australia; Queensland Brain Institute, University of Queensland, St. Lucia, Australia

**Keywords:** Drug-resistant epilepsy, Seizure onset zone (SOZ) localisation, Directed connectivity, Brain networks

## Abstract

Epilepsy affects over 50 million people worldwide, with approximately 30% experiencing drug-resistant forms that may require surgical intervention. Accurate localisation of the epileptogenic zone (EZ) is crucial for effective treatment, but how best to use intracranial EEG data to delineate the EZ remains unclear. Previous studies have used the directionality of neural activities across the brain to investigate seizure dynamics and localise the EZ. However, the different connectivity measures used across studies have often provided inconsistent insights about the direction and the localisation power of signal flow as a biomarker for EZ localisation. In a data-driven approach, this study employs a large set of 13 distinct directed connectivity measures to evaluate neural activity flow in and out the seizure onset zone (SOZ) during interictal and ictal periods. These measures test the hypotheses of “sink SOZ” (SOZ dominantly receiving neural activities during interictal periods) and “source SOZ” (SOZ dominantly transmitting activities during ictal periods). While the results were different across connectivity measures, several measures consistently supported higher connectivity directed towards the SOZ in interictal periods and higher connectivity directed away during ictal periods. Comparing six distinct metrics of node behaviour in the network, we found that SOZ separates itself from the rest of the network, allowing for the metric of “*eccentricity*” to localise the SOZ more accurately than any other metrics including “*in strength*” and “*out strength*.” This introduced a novel biomarker for localising the SOZ, leveraging the discriminative power of directed connectivity measures in an explainable machine learning pipeline. By using a comprehensive, objective, and data-driven approach, this study addresses previously unresolved questions on the direction of neural activities in seizure organisation and sheds light on dynamics of interictal and ictal activity in focal epilepsy.

## Author Summary

This study investigates how brain signals flow in patients with drug-resistant epilepsy. By analysing brain activity recordings, we found that the seizure origin (the epileptogenic zone or EZ) changes its role before and during seizures. Before a seizure, the EZ primarily receives signals, while during a seizure, it becomes a source, actively sending signals to other brain areas. To resolve the discrepancy in the literature, we tested a wide range of directed connectivity methods for tracking these signal flows and found that the metric of node “eccentricity” most accurately pinpoints the EZ. This research has the potential to improve surgical outcomes for people with epilepsy by providing a more precise way to locate the seizure origin.

## INTRODUCTION

More than 50 million people worldwide have epilepsy (Kwan & Brodie, 2000), and in about 30% of them, antiseizure medications cannot effectively control the disorder (Chen et al., 2018). In cases of focal epilepsy, where seizures originate from a specific part of one hemisphere, those with drug-resistant epilepsy may undergo presurgical evaluations to identify seizure-generating areas. This often involves intracranial electroencephalography (EEG) to delineate the epileptogenic zone (EZ), considered to be the sites primarily responsible for generating seizures (Lagarde & Bartolomei, 2024). If the clinical risk-benefit analysis is favourable, then the EZ can be surgically removed or disconnected through resection or laser ablation. Despite advancements in multimodal approaches like magnetic resonance imaging (MRI), EEG, and positron emission tomography (PET) scans, as well as extensive clinical expertise, accurate localisation of the EZ remains challenging and can hinder achieving seizure freedom (Vakharia et al., 2018).

Quantification methods have shown significant potential in localising the EZ by analysing intracranial EEG signals (Bartolomei et al., 2017; Bernabei et al., 2023; Gentiletti et al., 2022; Grinenko et al., 2018; Karimi-Rouzbahani & McGonigal, 2024). These methods typically focus on either the interictal or ictal time windows. In the ictal window, the most common epileptiform activities include low-voltage fast activity (LVFA; including narrow-band chirps), baseline slow-wave shifts, rhythmic spikes/spike waves, and preictal low-frequency spiking that are more dominant in seizure onset zone (SOZ^1^), where seizures are thought to originate from Bernabei et al. (2023). These features have been successfully extracted from signals and used for EZ localisation in previous studies (Di Giacomo et al., 2024; Grinenko et al., 2018; Lévesque et al., 2025). In the interictal window, traditional epileptiform characteristics that are quantified include interictal spikes/discharges and high-frequency oscillations (HFOs), with ongoing debate about which is more effective and ultimately possible increased predictive power by measuring their co-occurrence (Jahromi et al., 2024; Roehri et al., 2018; Shi et al., 2024; Zweiphenning et al., 2022). While clinical observations and animal models suggest spatial overlap between interictal and ictal neural activities to a variable degree (Avoli et al., 2006), the temporal balance between interictal and ictal states may depend on the directionality of activities measured using functional connectivity properties (Gunnarsdottir et al., 2022; Lagarde et al., 2018). Beyond the abovementioned epileptogenic patterns of ictal (e.g., LVFA, baseline shifts) and interictal (e.g., spikes and HFOs) activities, various other more complex and often nonlinear features have also been successfully used in localising the SOZ in both windows (Andrzejak et al., 2012; Mooij et al., 2020; Sato et al., 2019). In a recent work, we evaluated the performance of a large array of 34 distinct signal features in localising the EZ in both interictal and ictal windows. We showed that signal power and network-based connectivity features were amongst the most localising features and were amongst the most generalisable features across patients (Karimi-Rouzbahani & McGonigal, 2024).

While many traditional methods for EZ localisation focused on univariate or single-channel signal activity, there has been a shift towards multivariate/multichannel or network-based localisation (Gallagher et al., 2023; Gunnarsdottir et al., 2022; Johnson et al., 2023; Karimi-Rouzbahani et al., 2024a; Lagarde et al., 2018; Li et al., 2018, 2021). This approach aligns with the understanding of epilepsy as a network disorder (Kramer & Cash, 2012; Spencer, 2002) and has demonstrated better localisation performance compared with univariate methods in several studies (Balatskaya et al., 2020; Bernabei et al., 2022; Kini et al., 2019). These have established the connectivity measures as valuable biomarkers for EZ localisation both in the interictal (Corona et al., 2021, 2023; Englot et al., 2018; Goodale et al., 2020; Johnson et al., 2023; Lagarde et al., 2018; Rijal et al., 2023; Shah et al., 2019) and ictal (Liu et al., 2021; Rijal et al., 2023; Runfola et al., 2023; Schindler et al., 2007; Warren et al., 2010) periods. These studies focused on non-directed connectivity methods, which quantify the level of connectivity or interaction between areas but remain silent about the direction of activity flow. Specifically, they quantify the interaction without providing information about whether an area dominantly sends or receives neural activities from other connected areas.

The directionality of neural activity flows within and across epileptogenic networks can provide valuable insights such as where and how seizures are propagated and has become an area of growing interest within the field (Jiang et al., 2022; Li et al., 2023; Nahvi et al., 2023). One major hypothesis in epilepsy is the “interictal suppression hypothesis,” which posits that the EZ is inhibited by other brain areas in the interictal period (i.e., that this is why the brain does not seize constantly in subjects with epilepsy) and that seizures occur when this inhibition mechanism fails (Doss et al., 2024; Gunnarsdottir et al., 2022; Jiang et al., 2022; Johnson et al., 2023; Narasimhan et al., 2020; Paulo et al., 2022; Schevon et al., 2012; Vlachos et al., 2017). Previous studies have supported this hypothesis by showing inflow of spiking and HFOs (Matarrese et al., 2023; Wilke et al., 2009) in the interictal period as two well-established interictal epileptogenic patterns. Others have shown that the inflow is not limited to spiking activity and HFOs but can be seen in other more complex signal patterns as well (Gunnarsdottir et al., 2022; Jiang et al., 2022; Johnson et al., 2023; Narasimhan et al., 2020; Paulo et al., 2022; Vlachos et al., 2017) and has shown better localising performance than non-directed connectivity measures (Narasimhan et al., 2020). However, findings are nonunanimous with some studies showing dominant outflow of activity from the SOZ in the interictal period (Amini et al., 2011; Bettus et al., 2011; Lagarde et al., 2018; Wilke et al., 2009).

The directionality of epileptogenic activities in the ictal period (i.e., during seizures) has been suggested to dominantly flow out from the SOZ (Balatskaya et al., 2020; Courtens et al., 2016; Jung et al., 2011; Yang et al., 2018), proposing that SOZ is the source of epileptogenic activity (Gunnarsdottir et al., 2022; Jiang et al., 2022; Liou et al., 2020). Nonetheless, other studies have shown the opposite direction of neural activities dominantly *towards* the SOZ in the ictal period (An et al., 2020; Janca et al., 2021; Mao et al., 2016; Nahvi et al., 2023). Knowing the diverse patterns of epileptogenic activities during seizures (Lagarde et al., 2019), these studies tracked different aspects of neural activity including narrowband (Janca et al., 2021; Jiang et al., 2022; Li et al., 2023) and broadband (Courtens et al., 2016; Karimi-Rouzbahani et al., 2024b; Nahvi et al., 2023) activities. The evidence supporting both inward and outward epileptogenic activity relative to the SOZ questions the “SOZ suppression hypothesis” and complicates developing an understanding into the interaction between SOZ and other brain areas.

One main reason for the discrepancy between studies evaluating the direction of activity flow (connectivity) is the different directed connectivity methods used in different studies (Lagarde & Bartolomei, 2024; Lagarde et al., 2022) such as the complementary and well-established methods of directed transfer function (DTF) and Granger causality analysis (Eichler, 2006; Friston et al., 2014). As each directed connectivity method is mathematically distinct, they rely on distinct aspects of signals and their potential relationship (i.e., connectivity). For example, DTF measures the influence of one signal on another in the frequency domain using power analysis and Fourier transform, and the directed coherence (DCOH) method uses a spectral transfer matrix and normalises the inflow from one signal to another by their noise covariance (Baccalá & Sameshima, 2001). Such significant mathematical difference in the directed connectivity methods could impose different levels of sensitivity to connectivities across datasets (Plomp et al., 2014). Therefore, the development of more objective and data-driven approaches are required to determine the direction of activity towards and away from the SOZ (Doss et al., 2024; Lagarde & Bartolomei, 2024).

This study aims to establish the direction of neural activity in and out of the SOZ in an unbiased, data-driven, and objective pipeline. To that end, we used a large set of 13 mathematically distinct methods for quantifying directed connectivity used in the literature in the interictal and ictal periods. We then used network analysis metrics (also known as connectomes; Doss et al., 2024) including *in strength* and *out strength* to determine the dominant direction of broadband activity flow in and out of the SOZ. The aim is to see if the activities generally and dominantly flow towards or away from the SOZ and test the sink/source hypotheses in epilepsy with minimal effect of subjective method selection. Moreover, the knowledge about the directionality of neural activities in the interictal and ictal periods, if meaningful, can inform the development of automated EZ localisation methods. Specifically, if the SOZ were consistently at the receiving end of activity in the interictal period, this could be a valuable localising piece of information for automated algorithms. Therefore, to evaluate the localisation power of the directed connectivity methods in intracranial recording, we combined all directed connectivity methods to successfully localise the SOZ in both interictal and ictal periods.

## METHODS

### Dataset

This study uses a well-structured open-access intracranial dataset that brings together data from multiple centres (Bernabei et al., 2022; Kini et al., 2019). The dataset includes 57 patients who had been implanted with either subdural grid/strip (termed “electrocorticography” [ECoG]) or stereoelectroencephalography (SEEG) as their presurgical workup and subsequently treated with surgical resection or laser ablation. Two patients’ data were excluded from our analyses as one patient had no interictal and the other no ictal recording data for analyses. A summary of patients’ demographic information and the dataset is provided in Table 1 (see patients’ detailed demographics in Supporting Information Table S1). Clinically determined seizure onset channels were provided in the dataset. Each patient had two interictal recordings and between 1 and 5 (*mean* = 3.7) ictal recordings/seizures (110 interictal and 204 ictal recordings over all patients). The interictal data were selected from awake brain activities determined both by the selection of daytime recordings (8 am–8 pm) and the use of a custom non-rapid-eye-movement sleep detector (explained in detail in Bernabei et al., 2022). The interictal data were at least 2 hr before the beginning of a seizure and at least 2 hr after a subclinical seizure, 6 hr after a focal seizure and 12 hr after a generalised seizure, free of spikes and HFOs if possible, and not within the first 72 hr of recording to minimise immediate implant and anaesthesia effects. EZs/resected areas ranged from frontal to frontoparietal, mesiofrontal, temporal, mesiotemporal, parietal, and insular areas. Bad channels, as marked in the original dataset, were excluded from analyses. An average of 105.6 contacts (*std* = 38.04) per patient remained after bad channels were removed.

**Table 1.** Summary of patient demographics and dataset

Outcome	Therapy	Implant	Resection	Lesional/non	# channels recorded	% contacts within SOZ	Analysis data span (S)
Engel I (35)	Ablated (26)	SEEG (35)	Temporal (24)	Lesional (27)	MEAN = 104.96	MEAN = 12.87%	Ictal (whole seizure duration) (MEAN = 113 s, STD = 126 s)
Engel II–IV (20)	Resected (29)	ECOG (20)	Mesiotemporal (15)	Nonlesional (27)	STD = 35.1	STD = 11.1%	Interictal (MEAN = 120 s, STD = 0 s)
			Frontal (10)	n/a (1)			
			Mesiofrontal (2)				
			Insular (2)				
			Parietal (1)				
			Frontoparietal (1)				

### Preprocessing

The sampling frequencies of the signals varied across patients and ranged from 256 to 1024 Hz. We adjusted the sampling rate to 256 Hz across patients for analyses. We applied a 60-Hz notch filter to the data to remove line noise and minimise detection of spurious connectivities. To reduce the computational load, we only kept a maximum of 30 contacts per patient for analysis. In a random sampling procedure, we kept all the channels within the SOZ, and the other channels (remaining of 30) were randomly selected from non-SOZ contacts, which were inside the grey matter and at least 10 mm away from other contacts. The random sampling procedure was repeated 10 times for each patient, and the presented results for each patient are the average of the 10 sampling runs.

### Calculation of Directed Connectivity Measures

We selected a 2-min window of signal from each interictal recording (4 mins per patient) and a patient-specific length of signal from each ictal recording (from seizure onset to the termination of seizure). Within those windows, we selected three 2-s epochs of data for analysis. The three epochs were chosen to capture early, mid, and late dynamics of the signals within each recording. Specifically, in the 2-min interictal window, the first (0–2 s), middle (from 59 s to 61 s), and last 2 s (118–120 s) were selected as early, mid, and late time epochs, respectively. In the ictal period, the early and late epochs were separated by the length of the seizure (i.e., variable), while the mid epoch was selected as the 2-s epoch halfway between the early and late epochs. Our choice of 2-s epochs was to select a midrange epoch compared with previous studies that used a wide range of epochs from 0.25 s to 10 min in the interictal period (Balatskaya et al., 2020; Mooij et al., 2020; Sato et al., 2019) with temporally unchanging results (Gunnarsdottir et al., 2022) and from 20 to 60 s in the ictal period (Li et al., 2018; Runfola et al., 2023). Another reason for this choice was to avoid the potential nonstationarity of the signals that could have had larger impacts with longer analysis windows and could differentially affect specific connectivity measures that are sensitive to signal stationarity, but not all (Table 2).

**Table 2.** Summary of directed connectivity measures demographics and data

Connectivity measure	Operation	Strengths	Weaknesses	Assumptions
Additive Noise Model (ANM) (Hoyer et al., 2008)	Models influence as additive noise.	Linear, computationally efficient.	Limited to linear relationships, sensitive to noise and model order.	Assumes stationary noise terms.
Information-Geometric Causal Inference (IGCI) (Janzing et al., 2012)	Exploits geometric concepts in information theory.	Nonlinear, robust to noise and bias.	Computationally demanding, requires sufficient data.	Can be affected by nonstationarities that significantly alter probability distributions.
Conditional Distribution Similarity Fit (CDS) (Cliff et al., 2023)	Compares conditional probability distributions.	Captures nonlinear relationships, robust to noise.	Computationally demanding, requires careful parameter selection.	Can be affected by nonstationarities that significantly alter probability distributions.
Regression Error-Based Causal Inference (RECI) (Blöbaum et al., 2018)	Compares prediction errors.	Simple to implement, applicable to various data types.	Sensitive to model misspecification, noise, and outliers.	Assumes linear relationships and stationary data.
Causally Conditioned Entropy (CCE) (Cliff et al., 2023)	Exploits information theory to infer causal directions.	Captures nonlinear relationships, applicable to various data types.	Computationally demanding, sensitive to data quality and assumptions.	Can be sensitive to nonstationarities that violate underlying assumptions.
Directed Information (DI) (Massey, 1990)	Quantifies directed transfer of information between time series.	Model-free, captures nonlinear relationships.	Sensitive to noise and data length, computationally demanding.	Assumes stationarity of the time series.
Group Delay (GD) (Hannan & Thomson, 1973)	Infers directionality based on time delays.	Simple to compute, provides insights into temporal dynamics.	Sensitive to noise and artefacts, assumes linear relationships and constant time delays.	Assumes stationarity and linearity.
Phase Slope Index (PSI) (Nolte et al., 2008)	Measures consistency of phase slope across frequencies.	Less sensitive to volume conduction effects.	Sensitive to noise and artefacts, may be less robust in strong nonlinearities.	Can be affected by some types of nonstationarity.
Directed Transfer Function (DTF) (Eichler, 2006)	Estimates directed influences in multivariate autoregressive models.	Captures frequency-specific influences, provides a comprehensive picture.	Sensitive to model order selection and noise, influenced by volume conduction.	Assumes linearity and stationarity of time series.
Directed Coherence (DCOH) (Baccalá & Sameshima, 2001)	Measures directed influence between two signals in the frequency domain.	Captures frequency-specific influences, relatively robust to volume conduction effects.	Sensitive to noise and artefacts, may be influenced by common sources of noise.	Assumes linearity of relationships.
Partial Directed Coherence (PDC) (Baccalá & Sameshima, 2001)	Estimates directed influences while controlling for other sources.	Captures frequency-specific influences, disentangles direct and indirect influences.	Sensitive to model order selection and noise, influenced by model assumptions.	Assumes linearity and stationarity of time series.
Spectral Granger Causality (SGC) (Friston et al., 2014)	Extends Granger causality to the frequency domain.	Provides frequency-specific information about causal influences.	Sensitive to noise and model order selection, assumptions of linearity and stationarity may be violated.	Assumes linearity and stationarity of time series.
Linear Model Fit (LMFIT) (Cliff et al., 2023)	Fits linear models to data.	Computationally efficient, widely used.	Assumes linear relationships, sensitive to outliers and noise.	Assumes linearity and stationarity of relationships.

We used the open-source Python toolbox called PySpi (Cliff et al., 2023), which implemented the largest set of directed and nondirected connectivity measures for time series (here, intracranial EEG channels). We used the 13 available directed connectivity measures implemented in the toolbox for this work to follow an unbiased data-driven approach in analysis. Majority of the directed connectivity measures used in intracranial data analysis are amongst these 13 measures. We categorised the measures into “information theory” (*n* = 6), “frequency-domain” (*n* = 6), and “time-domain” (*n* = 1) methods. For the frequency-domain measures, we used the full frequency range of 0–128 Hz to obtain a broadband index rather than focusing on a narrow frequency band. This provides general and objective results. The PySpi toolbox calculates the measures over 125 uniformly sampled bins across the 0- to 128-Hz frequency range before averaging them. The upper bound of 128 Hz was selected according to the Nyquist theorem, stating that highest undistorted frequency is half the signal sampling frequency (i.e., 256 Hz here).

For more information about each connectivity measure, the reader is advised to study the references cited for each measure and Supporting Information Text S1. Table 2 provides a summary of the measures.

### Node Metrics

To characterise the role and behaviour of each node in the brain network, we used several network analysis metrics (i.e., connectomics). Specifically, in network analysis, each network is composed of **nodes** that are the electrode contacts here and **links** that are the (assumed) internode connections. Using the open-source Brain Connectivity Toolbox (Rubinov & Sporns, 2010), we extracted six node metrics to evaluate the node behaviour in the network:

*In strength* is the sum of inward link weights (connectivity values). Nodes with higher *in strength* are influential receivers within the network, as they accumulate a significant amount of incoming influence, resources, or interactions from other nodes.

*Out strength* is the sum of outward link weights. Nodes with higher *out strength* are influential senders within the network, as they contribute a significant amount of outgoing influence, resources, or interactions to other nodes.

*First passage time* is the expected number of steps it takes a random walker to reach one node from another. Nodes with higher first passage times are often located on the periphery, farther away from central or densely connected regions.

*Clustering coefficient* is the fraction of triangles around a node and is equivalent to the fraction of node’s neighbours that are neighbours of each other. Nodes with higher clustering coefficients are typically located in densely connected neighbourhoods. These nodes have many connections to neighbouring nodes, forming cohesive groups or communities.

*Eccentricity* is the maximal shortest path length between a node and any other node. Nodes with higher eccentricity are typically located on the periphery of the network. They are farther away from the central core or densely connected regions.

*Betweenness centrality* is the fraction of all shortest paths in the network that contain a given node. Nodes with higher betweenness centrality often serve as bridges or connectors between different clusters, communities, or groups within the network. They lie on many of the shortest paths connecting nodes in different regions. Nodes with lower betweenness centrality are typically located on the periphery of clusters or communities within the network. They have fewer connections to other nodes and are less likely to lie on shortest paths between nodes.

### Sink and Source SOZ Hypotheses

The fundamental “suppression hypothesis” suggests that the SOZ is inhibited by other brain areas in the interictal period and that seizures occur when this inhibition mechanism fails (Doss et al., 2024; Gunnarsdottir et al., 2022; Jiang et al., 2022; Johnson et al., 2023; Narasimhan et al., 2020; Paulo et al., 2022; Schevon et al., 2012; Vlachos et al., 2017), leading to the outflow of epileptic activities from the SOZ. Here, we built on these recent works and used a large set of directed connectivity measures and novel network analyses to quantitatively test this fundamental hypothesis. Sink contacts/areas were defined here as those that showed significantly higher *in strength* than other contacts, and source contacts/areas were those that showed significantly higher *out strength* than other contacts. Here, we checked to see if *in strength* was higher in SOZ than non-SOZ contacts to support the “sink SOZ” in the interictal period and if *out strength* was higher in SOZ than non-SOZ areas to support the “source SOZ” in the ictal period.

### Multivariate Pattern Classification for SOZ Localisation

**Figure 1.**
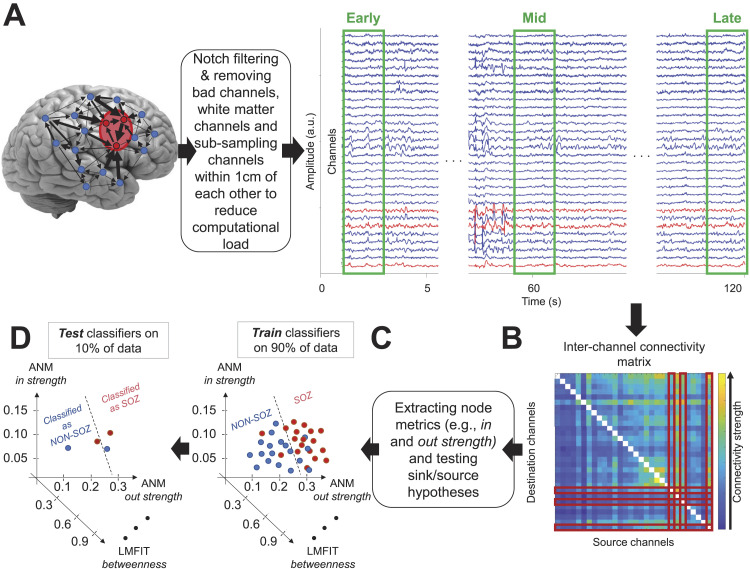
The proposed localisation pipeline. (A) Recorded signals were notch-filtered and three 2-s epochs of data were extracted for analyses. (B) An intercontact directed connectivity matrix reflecting connectivity strengths (colour-coded) and direction was developed, with red squares indicating SOZ contacts (columns and rows represent source and destination areas, respectively). (C) Several node metrics were extracted from the connectivity matrices to assess each node’s behaviour in the network. (D) Machine learning classifiers were used to distinguish contacts within and outside the SOZ in a 10-fold cross-validation process.

We employed a standard multivariate pattern classification approach to localise the SOZ, distinguishing contacts within the SOZ from those outside it (non-SOZ; Karimi-Rouzbahani & McGonigal, 2024). The term multivariate used here refers to the multiple node metrics extracted from connectivity measures fed to the classifiers for the discrimination of contacts (i.e., the 78-dimensional feature set consisting of six node metrics per 13 connectivity measures). Initially, we computed interchannel directed connectivity values. Subsequently, we calculated the six aforementioned node metrics for each contact based on the connectivity matrix, which has a size of *N* × *N*, where *N* represents the number of nodes or contacts. These metrics were then concatenated and utilised as features for the classifiers. The classification performance gauged the discriminability of SOZ and non-SOZ contacts using directed connectivity measures, assessed by the area-under-the-curve (AUC) metric for comprehensive, threshold-free classification performance. Consistent with recent localisation studies (Jiang et al., 2022; Karimi-Rouzbahani & McGonigal, 2024), we employed a Random Forest classifier in MATLAB using the TreeBagger function. The training data were normalised to ensure consistent feature scaling (separately for training and testing sets to avoid any potential leakage of information from the testing to the training set). A 50-tree decision-tree ensemble was constructed. Each contact was imported as an observation, and each node metric was extracted from each connectivity measure as a feature into the classifiers. Decision-tree classifiers are suitable for nonlinear feature classifications and offer insights into feature contributions. We used the OOBPermutedPredictorDeltaError option to assess the “contribution” of each feature in the classification process. This approach clarifies the contribution of each feature by permuting the contact labels (i.e., SOZ vs. non-SOZ) for each feature separately and assessing its impact on performance, where contribution is inversely proportional to performance drop. For classification, we conducted separate analyses within interictal and ictal time windows for each patient, employing a 10-fold cross-validation procedure. This procedure was applied individually for each recording data from ictal and interictal packets and their combinations. While, on average, there was a comparable number of SOZ and non-SOZ contacts for each patient in the analyses, there was a substantial variance (*std* = 11.1%). To make sure the classification performance was not affected by class imbalance, we balanced the number of observations across our classes through an up-sampling procedure. We increased the number of observations for the class with fewer observations/contacts, repeating each classification of data 1,000 times before averaging the results. For the up-sampling, we simply repeated the data from the class with fewer samples (contacts) so that both classes had a perfectly equal number of samples. The up-sampling procedure was done only for the training set within each cross-validation fold to avoid leakage to the test set. We generated chance-level performances by shuffling (SOZ/non-SOZ) contact labels 1,000 times and recalculating the classification performance, resulting in 1,000 chance-level classification outcomes against which we assessed the significance of our true classification performances. The SOZ localisation pipeline is shown in Figure 1.

### Statistical Analysis

Except for correlation analyses, all other statistical tests were performed using Bayes Factor (BF) analyses. In all BF analyses, we interpreted the levels of BF evidence more strictly than conventional thresholds (Dienes, 2014; Lee & Wagenmakers, 2005): BFs above 10 and below 1/10, were considered evidence for the alternative and null hypotheses, respectively. BFs falling between 1/10 and 10 were regarded as providing insufficient evidence either way, indicating that no conclusions could be drawn about effects and interactions between variables. Specifically, we used BFs in four different statistical analysis scenarios.

First, we used BF *t* tests to compare the level of node metrics between SOZ and non-SOZ electrode contacts for different connectivity measures. In this case, we assessed the evidence for the alternative (i.e., different; H1) and the null (i.e., no difference; H0) hypotheses.

Second, we used BF *t* tests to compare the level of localisation performance (AUCs) against chance/null performance (obtained through the randomisation explained above). In this case, we assessed the evidence for the alternative (i.e., different from chance; H1) and the null (i.e., no difference; H0) hypotheses.

Third, we used BF *t* tests to compare the contribution of different pairs of connectivity measures × node metrics with the localisation performance. In this case, we assessed the evidence for the alternative (i.e., different; H1) and the null (i.e., no difference; H0) hypotheses. When comparing the contribution of each connectivity measure, we concatenated its node metrics, and when evaluating the contribution of each node metric, we concatenated all connectivity measures. We concatenated all patients’ data in both analyses.

Finally, we used BF analysis of variance (ANOVA) to evaluate the main effects of surgery outcome (Engel I/Engel II–IV), region of resection (Frontal/Temporal/Mesial Temporal, FRT/TPRMTL), pathology (lesional/nonlesional), and recording modality (SEEG/ECoG). In this analysis, these four factors served as independent variables, with localisation performance (AUC) as the dependent variable. To ensure statistical power in ANOVA, we excluded the patients with insular (*n* = 2), frontoparietal (*n* = 1), parietal (*n* = 1), and mesiofrontal (*n* = 2) resections, where the sample size was insufficient for statistical analyses. Priors for all BF analyses were determined based on Jeffrey-Zellner-Siow priors (Jeffreys, 1998; Zellner & Siow, 1980), which are derived from the Cauchy distribution based on the effect size initially calculated in the algorithm using *t* tests (Rouder et al., 2012).

We used *Pearson* correlation analysis to evaluate the level of dependence between variables including between node in and out strengths in different time periods (interictal vs. ictal).

## RESULTS

**Figure 2.**
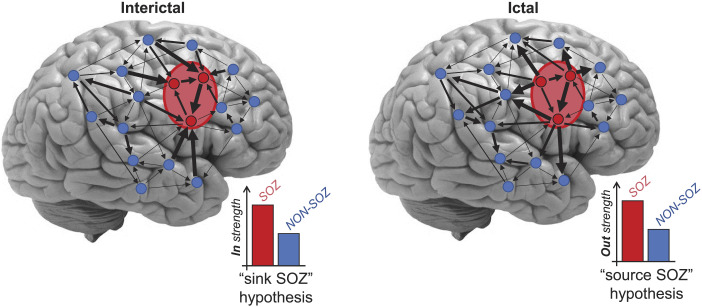
“Sink” and “source” SOZ hypotheses. Sample implanted electrodes with arrows indicating the strength of activity flows/connectivity (thicker arrows represent stronger connections) between electrode contacts. The “SOZ sink” hypothesis suggests a higher *in strength* for SOZ than non-SOZ contacts in the interictal period, and the “SOZ source” hypothesis suggests a higher *out strength* in SOZ than non-SOZ contacts in the ictal period.

We tested the sink-source hypotheses as explained (Figure 2) and observed that there was higher connectivity towards the SOZ in the interictal period and higher connectivity going out of the SOZ in the ictal period. We also used our large set of 13 distinct measures of directed connectivity and node metrics to successfully localise the SOZ using machine learning classifiers.

### Strength of Neural Activity Flow In and Out of the SOZ

**Figure 3.**
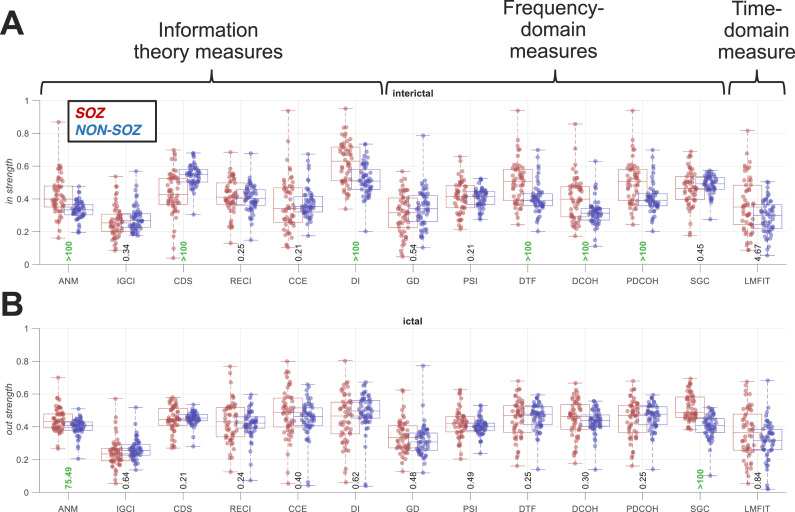
*In strength* in the interictal (A) and *out strength* in the ictal (B) period across connectivity measures. Connectivity measures are categorised into information theory, frequency-domain, and time-domain measures. Results are separated for the SOZ (red) and non-SOZ (blue) contacts, with each dot showing data for one patient. Box plots show the distribution of data, its quartiles, and its median, and whiskers indicate the maximum and minimum of the data across patients. Each dot indicates the data from one patient. Numbers below the bars indicate Bayesian evidence (BF > 10 indicated in green) for the difference between SOZ and non-SOZ data.

In the interictal period, across the 13 directed connectivity measures, there was evidence (BF > 10) for higher *in strength* in SOZ than non-SOZ areas for five connectivity measures (ANM [additive noise model], DI [directed information], DTF, DCOH, and PDCOH [partial DCOH]), and there was evidence (BF > 10) for higher *in strength* in non-SOZ than SOZ areas only for the conditional distribution similarity fit (CDS) connectivity measure (Figure 3A). There was insufficient evidence (0.1 < BF < 10) either way for the rest of the connectivity measures. In the ictal period, there was evidence (BF > 10) for higher *out strength* in SOZ than non-SOZ areas for ANM and spectral Granger causality (SGC) connectivity measures, respectively. There was insufficient evidence (0.1 < BF < 10) either way for the rest of the connectivity measures (Figure 3B). Separate analyses of seizure-free (Engel I) and non-seizure-free (Engel II–IV) patients showed similar patterns of results (Supporting Information Figure S1).

The results above were obtained by averaging the results from *early*, *mid*, and *late* epochs (time windows) of the interictal and ictal periods (Figure 1B). To evaluate potential temporal variability in connectivity, we also evaluated *in strength* and *out strength* for each individual 2-s epoch separately (Supporting Information Figure S2). The patterns of *in strength* were relatively similar across the three interictal windows (cf., Figure 3A). The patterns of *out strength* were also similar across the three ictal windows and resembled the time-averaged results (cf., Figure 3B).

While higher *in strength* in SOZ than non-SOZ areas (e.g., in the interictal period; cf., Figure 3A) does not necessarily correspond to higher *out strength* in non-SOZ than SOZ areas, we tested this opposite nonhypothesised effect as well to ensure that we are not overlooking a relevant effect (Supporting Information Figure S3). In the interictal period, there was evidence (BF > 10) for higher *out strength* in non-SOZ than SOZ areas for the CDS connectivity measure only. However, there was evidence (BF > 10) for higher *out strength* in SOZ than non-SOZ areas for ANM and SGC, respectively. In the ictal period, there was evidence (BF > 10) for higher *in strength* in non-SOZ than SOZ areas for the CDS only, but also evidence (BF > 10) for higher *in strength* in SOZ than non-SOZ areas. Therefore, the *out strength* during interictal period and the *in strength* during the ictal period provided inconsistent results across connectivity measures to support opposite activity flows.

Together, these results show that distinct measures of connectivity show different results. Nonetheless, 5 of 13 connectivity measures supported the “sink SOZ” hypothesis in the interictal period. Similarly, but less strongly than in the interictal period, two connectivity measures supported the “source SOZ” hypothesis in the ictal period. These results were relatively stable within the interictal and ictal periods. Amongst the 13 connectivity measures evaluated, the ANM measure supported both hypotheses.

### Switching of Activity Direction From the Interictal to Ictal Period

**Figure 4.**
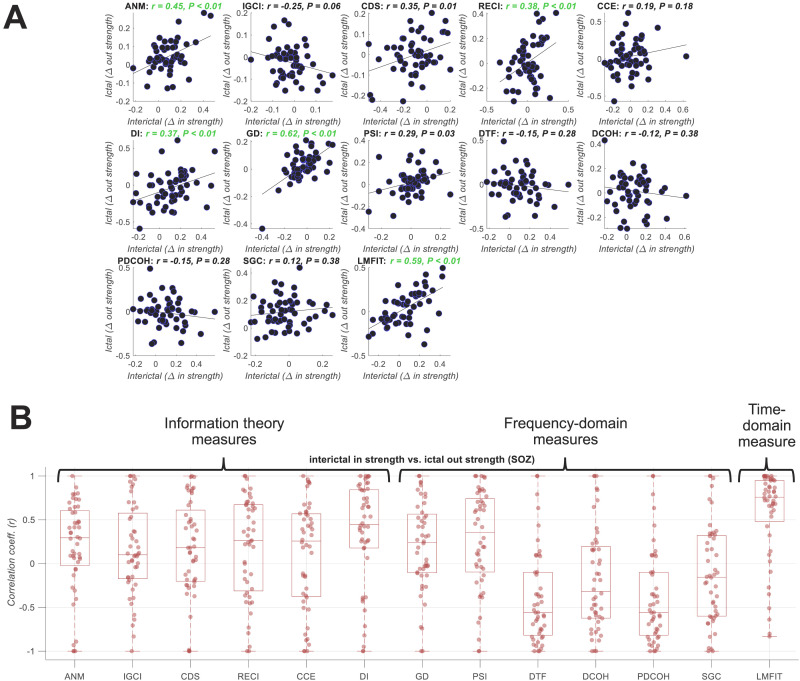
SOZ switch from a receiver to a sender of activity from interictal to ictal period. (A) *Pearson* linear correlations between interictal and ictal effect sizes across patients, with each dot showing data from one patient (i.e., effect size = difference between SOZ and non-SOZ contacts in terms of *in strength* in the interictal and *out strength* in the ictal period). Correlations and the corresponding *p* values are shown on top of each panel (significant results green: *p* < 0.01) with the slant line showing the best linear fit to the data. (B) *Pearson* linear correlation between interictal and ictal effect sizes across contacts with each dot showing cross-contact averaged results for an individual patient (i.e., effect size = difference between *in strength* in the interictal and *out strength* in the ictal period for each contact).

As the above results were interpreted at a group level, the higher interictal *in strength* in SOZ compared with non-SOZ and the higher ictal *out strength* in non-SOZ compared with SOZ could have come from distinct subsets of patients. To assure that the tendency of switching roles between sink and source is consistent for each patient (Doss et al., 2024), we evaluated the correlation between effect sizes in the interictal and ictal periods (Figure 4A; effects sizes: Δ = *in strength*_*SOZ*_ − *in strength*_*non-SOZ*_ in interictal and Δ = *out strength*_*SOZ*_ − *out strength*_*non-SOZ*_ in ictal data). Majority (8 of 13) of connectivity measures showed positive correlations between the direction of effects across the interictal and ictal periods over patients, with five reaching significance at *p* < 0.01 (*Pearson* correlation). These significant correlations suggested that the same patients with higher *in strength* in SOZ also showed higher *out strength* in SOZ contacts in the ictal data. The correlations were nonsignificantly negative for the rest of the five connectivity measures.

To check if this switching of roles was also consistent at the individual contact level, we performed an additional correlation-based analysis. The level of correlation between interictal *in strength* and ictal *out strength* across SOZ contacts, within each individual patient (Figure 4B, top panel), looked like the cross-patient analysis (cf., Figure 4A) and showed positive correlations for nine of the connectivity measures while the rest showed negative correlations. This suggests that changes in signal characteristics and connectivity patterns from the interictal to ictal period impacts distinct connectivity measures differently. The consistent positive correlations across the nine connectivity measures supported that the contacts with higher interictal *in strength* also showed a higher ictal *out strength*. However, the four measures with nonsignificant negative correlations, which were all frequency-domain measures, captured a different transition: The contacts with higher interictal *in strength* showed a lower ictal *out strength*.

Together, these results suggest that SOZ areas switch roles from dominantly receiving neural activity in the interictal period to dominantly transmitting them in the ictal period. This was captured by majority (9 of 13) of connectivity measures.

### Separation of the SOZ From the Rest of the Network

Having tested the “sink and source SOZ” hypotheses, we then tested to see if other node metrics/behaviours could distinguish between SOZ and non-SOZ areas. These metrics can show if SOZ and non-SOZ play different parts in the brain network. Specifically, we compared SOZ and non-SOZ contacts using four additional node metrics including *first passage time*, *clustering coefficient*, *eccentricity*, and *betweenness*.

**Figure 5.**
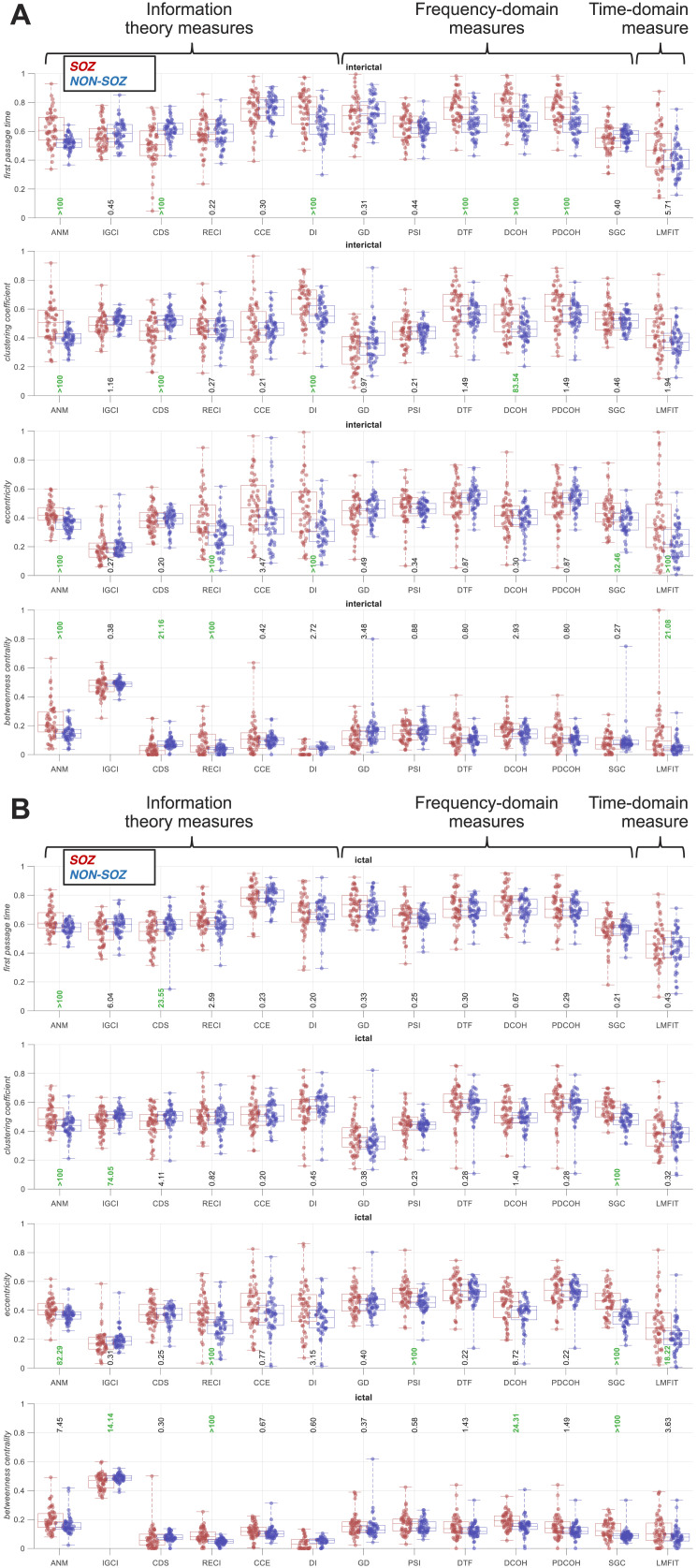
*First passage time*, *clustering coefficient*, *eccentricity*, and *betweenness centrality* in the interictal (A) and the ictal (B) periods across connectivity measures. Connectivity measures are categorised into information theory, frequency-domain, and time-domain measures. Results are separated for the SOZ (red) and non-SOZ (blue) contacts, with each dot showing data from one patient. Box plots show the distribution of data, its quartiles, and its median, and whiskers indicate the maximum and minimum of the data across patients. The numbers below (above for betweenness centrality) the bars indicate Bayesian evidence (BF > 10 indicated in green) for the difference between SOZ and non-SOZ data.

In the interictal period, there was evidence (BF > 10) for higher *first passage time* in SOZ than non-SOZ for five (ANM, DI, DTF, DCOH, and PDCOH) and higher *eccentricity* for five (ANM, RECI [regression error-based causal inference], DI, SGC, and LMFIT [linear model fit]) connectivity measures (Figure 5A). *Clustering coefficient* and *betweenness centrality* showed less-consistent results across connectivity measures. There was evidence (BF > 10) for higher *clustering coefficient* in SOZ than non-SOZ for three connectivity measures (ANM, DI, and DCOH) but also evidence (BF > 10) for lower *clustering coefficient* in SOZ than non-SOZ for CDS. There was evidence (BF > 10) for higher *betweenness centrality* in SOZ than non-SOZ for three connectivity measures (ANM, RECI, and LMFIT) but also evidence (BF > 10) for lower *betweenness centrality* in SOZ than non-SOZ for CDS.

In the ictal period, results were less consistent across measures in terms of *first passage time* and *clustering coefficient*, with two connectivity measures higher for SOZ and one higher for non-SOZ (Figure 5B). There was evidence (BF > 10) for higher *eccentricity* in SOZ than non-SOZ for five connectivity measures (ANM, RECI, PSI [phase slope index], SGC, and LMFIT) similar to the interictal period. There was evidence (BF > 10) for higher *betweenness centrality* in SOZ than non-SOZ for three connectivity measures (RECI, DCOH, and SGC) but also evidence (BF > 10) for lower *betweenness centrality* in SOZ than non-SOZ for information-geometric causal inference (IGCI).

Together, the relative consistency and non-opposing results across connectivity measures in terms of *first passage time* in the interictal data suggest that SOZ areas had more complex and prolonged interactions (i.e., increased *first passage times*) with other areas than non-SOZ, potentially due to abnormal neural activity. In the same data, a higher *eccentricity* in SOZ than non-SOZ contacts suggests that the SOZ was positioned distantly from the rest of the network, indicating a more peripheral position or a more complex and distributed network structure for the SOZ than the non-SOZ.

### Localisation of SOZ Using Network Metrics

**Figure 6.**
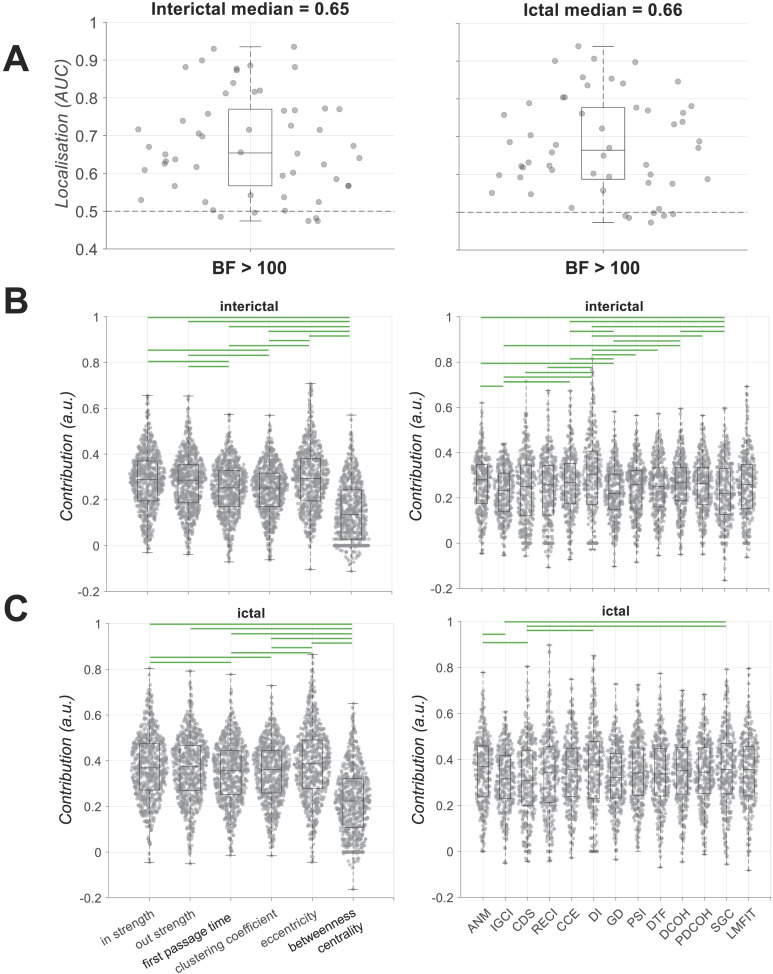
Classification of SOZ and non-SOZ contacts (localisation). (A) AUC of classification performance for interictal and ictal data. Box plots show the distribution of data, its quartiles, and its median, and whiskers indicate the maximum and minimum of the data over patients. Each dot indicates the data from one patient. Numbers below the bars indicate Bayesian evidence for the difference between the true and chance performance. Horizontal dashed line refers to theoretical chance-level classification (0.5). Contribution of each connectivity measure and node metric to the classification performance is shown in the interictal (B) and ictal (C) period. Contributions are calculated using random permutation in the classification. Green horizontal lines indicate evidence (BF > 10) for difference between contributions.

Finally, we used our set of six node metrics extracted from the 13 connectivity measures to localise the SOZ. There was evidence (BF > 10) for above-chance (i.e., AUC > 0.5) localisation of the SOZ in both interictal and ictal periods at the group level (Figure 6A). At the individual patient level, the performance varied across patients from around chance level of 0.5 to above 0.9. These results showed that, for some patients, there was enough information in the directed connectivity measures to predict if a node was part of the SOZ or not. These results also showed that interictal activity could also provide as much localisation power as the ictal activity, which is dominantly used in clinical practice. There was insufficient evidence (0.1 < BF < 10) for any effects of these demographic variables (surgery outcome [Engel I/Engel II–IV], region of resection [FRT/TPRMTL], pathology [lesional/nonlesional] on the localisation performance in either interictal or ictal data [Supporting Information Figure S4; BF ANOVA *t* test]).

We then asked if there was systematic temporal variation across the different recordings (i.e., time windows of interictal and ictal/seizure data) and analysis epochs (i.e., early, mid, and late). To perform this analysis, classifiers were trained and tested within each individual recording and epoch. While there was no evidence for difference between performances across interictal epochs (BF < 10; Supporting Information Figure S5A), the localisation performance was higher in early than mid and late epochs of several ictal recordings (BF > 10; Supporting Information Figure S5B). The latter can be explained by a larger separation of SOZ from non-SOZ areas at the onset of seizures. Please note that while the early epoch of data always contained the first two seconds of the ictal activity, the mid and late epochs did not match across patients as the length of seizures differed.

We then evaluated the contribution of each connectivity measure and node metric to the localisation performance. In the interictal period (Figure 6B), there was evidence (BF > 10) for higher contribution of *eccentricity* than *first passage time* and *clustering coefficient* and lower contribution from *betweenness centrality* than the other five node metrics. The median contributions of other node metrics varied in the range between the medians of *betweenness centrality* and *eccentricity*. There was evidence (BF > 10) for higher contributions of DI and ANM and lower contribution of SGC than several other node metrics. In the ictal period (Figure 6C), we observed relatively similar contributions of node metrics and connectivity measures. Specifically, node *eccentricity* and *betweenness centrality* showed the highest and lowest contributions amongst other node metrics. There was also evidence (BF > 10) for higher contribution of DI and ANM than other connectivity measures.

Together, these results show that the node metric of *eccentricity*, which reflects how peripheral a node’s position was in the network, had even higher localisation power than *in strength* and *out strength*. We also observed that the connectivity metrics of DI and ANM provided the highest localisation power. These results were consistent across both interictal and ictal periods. We also observed that localisation was improved when done separately for each time window and was higher in early than later epochs of the ictal data. These results suggest that, there are subtle variations in node connectivity patterns (possibly more dominant in *eccentricity*), which can change the discriminability of SOZ from non-SOZ over time.

## DISCUSSION

This work was aimed towards two goals. First, to test if the SOZ in people with focal epilepsy dominantly receives neural activities in the interictal (resting baseline state) period (“sink SOZ” hypothesis) and dominantly transmits the activities in the ictal (seizure) period (“source SOZ” hypothesis). To that end, we utilised a data-driven approach and recruited a set of 13 directed connectivity measures along with six metrics of node behaviour in the network. We found that not all connectivity measures supported the above hypotheses. Nonetheless, we found evidence across several connectivity measures supporting these hypotheses and almost no evidence for an opposite pattern. These measures showed that SOZ dominantly received neural activities in the interictal and transmitted them in the ictal period, supporting the idea of seizure suppression and propagation, respectively. Second, this study evaluated the predictive power of node metrics extracted from the abovementioned connectivity measures in localising the SOZ. To that end, we utilised the power of explainable machine learning classifiers to successfully discriminate contacts inside from those outside the SOZ. This work makes several contributions to our understanding of epilepsy and how hypothesis-driven biomarkers can localise the SOZ.

One critical aspect of our study is its use of BF analysis, which allowed us to test if there was evidence “for,” “against,” or “no evidence either way” about our “sink SOZ” and “source SOZ” hypotheses (e.g., sink SOZ: in strength in SOZ > non-SOZ in interictal period and “source SOZ”: out strength in non-SOZ > SOZ in ictal period). This is a big advantage to the common frequentist statistical tests (e.g., Wilcoxon’s or Student’s *t* tests) where one tests if a hypothesis is “true” or “not” in a binary fashion. We also showed the level of evidence for each hypothesis and used strict thresholds for interpreting BF evidence. While five connectivity measures showed evidence for the “sink SOZ” hypothesis, only one connectivity measure (CDS) showed evidence against the “sink SOZ” in the interictal period, and the rest of the seven connectivity measures showed no evidence either way. In the ictal period, two connectivity measures showed evidence for the “source SOZ” hypothesis, no connectivity measures showed evidence against it, and the other 11 connectivity measures did not show any evidence either way. Accordingly, our BF analyses of the results showed variations across measures but tended to provide support for the “sink SOZ” in the interictal and “source SOZ” in the ictal period. Importantly, we did not see unanimous results across all connectivity measures, which was expected as different connectivity measures inherently differed, and one main aim of this study was to reveal their difference in behaviour. This suggests that future studies should consider adopting data-driven approaches to produce replicable findings.

Earlier studies evaluated the directionality of signals in the interictal and ictal periods. In interictal data, some studies have suggested a leading role (higher outgoing signals) for the EZ (Bettus et al., 2008; Lagarde et al., 2018; Varotto et al., 2012), whereas others suggested the opposite (Gunnarsdottir et al., 2022; Jiang et al., 2022; Narasimhan et al., 2020; Paulo et al., 2022; Vlachos et al., 2017). Similar discrepancy exists in studies that used ictal data, with some studies suggesting a leading role for the EZ areas (Balatskaya et al., 2020; Courtens et al., 2016; Jung et al., 2011; Yang et al., 2018) and others providing evidence for the opposite (An et al., 2020; Janca et al., 2021; Mao et al., 2016; Nahvi et al., 2023). One important reason behind these discrepant results could be the variation in the methods used to measure directed connectivity (Doss et al., 2024; Lagarde & Bartolomei, 2024; Lagarde et al., 2022). Basically, distinct connectivity methods rely on distinct signal features to quantify connectivity. As we categorised these methods (cf., Figure 3), some methods rely on the complexity, randomness, or the predictability of signal samples (information theory methods), whereas some rely on frequency-domain representation of signals (frequency-domain methods) and others simply rely on one-to-one mapping of time samples across areas (time-domain measures, e.g., LMFIT; Cliff et al., 2023). Even different methods within each category work differently. For example, while ANM detects causal relationship which are assumed to be additive with independent noise, IGCI can detect more complex nonadditive relationships. Therefore, it is not surprising to detect the directed connectivity using one method but not the other.

Building on the recent developments in neural decoding (Karimi-Rouzbahani, 2024; Karimi-Rouzbahani, Shahmohammadi, et al., 2021; Karimi-Rouzbahani & Woolgar, 2022) and connectivity analyses (Cliff et al., 2023; Karimi-Rouzbahani, Ramezani, et al., 2021; Karimi-Rouzbahani et al., 2022) and trying to avoid subjective analysis (Pavlov et al., 2021; Trübutschek et al., 2024), this study adopts a data-driven and objective approach to test the direction of neural activity flow towards and away from the SOZ, which has been lacking in previous studies that tend to select a priori methods of connectivity analysis (Lagarde & Bartolomei, 2024). We showed that several connectivity measures showed higher *in strength* towards SOZ than non-SOZ areas in the interictal and higher *out strength* from SOZ than non-SOZ in the ictal period. These results supported a switching role for the SOZ that not only supports the hypotheses of “sink SOZ” in the interictal (Gunnarsdottir et al., 2022) and “source SOZ” in the ictal period (Schindler et al., 2007) but also serves as a biomarker for localising SOZ. Interestingly, we observed a higher consistency across connectivity measures in the interictal than ictal period. This might suggest that while the inflow of activity in the interictal period might be reflected in a wider range of activity patterns as captured by a higher number of connectivity measures, the outflow of neural activity might be confined to a limited range of activity patterns (Lagarde et al., 2019). This is supported by our observation of less cross-patient generalisable epileptogenic patterns in the interictal than ictal periods (Karimi-Rouzbahani & McGonigal, 2024).

Only a few studies have evaluated directed connectivity during both interictal and ictal periods in the same patient population. For example, the PDCOH method applied to patients with type II focal cortical dysplasia showed a higher *out density* (defined as the ratio between the sum of node degrees and the total number of connections in the network) in the lesion and SOZ areas than non-SOZ areas supporting the “source SOZ” hypothesis in the ictal data, but did not find evidence to support “sink SOZ” in the interictal data (Varotto et al., 2012). On the other hand, phase transfer entropy along with node metrics applied to a sample of 43 temporal lobe epilepsy patients showed higher *out/in degree ratio* in EZ than non-EZ areas consistently through interictal and ictal data, supporting “source SOZ” hypothesis in the ictal period (Wang et al., 2017). A more recent study used both interictal and ictal data and supported the “sink SOZ” hypothesis in the interictal and “source SOZ” hypothesis in the ictal data using DTF measure (Jiang et al., 2022). Finally, using a novel source-sink index obtained from both interictal and ictal activities, both the interictal sinking and ictal sourcing behaviours were observed for SOZ (Gunnarsdottir et al., 2022). In our work, DTF supported “sink SOZ” in the interictal data but showed insufficient evidence (0.1 < BF < 10) for “source SOZ” in the ictal data (cf., Figure 3A). As these previous studies only used one (Varotto et al., 2012) or a couple (Jiang et al., 2022; Narasimhan et al., 2020; Vlachos et al., 2017) of connectivity measures, there is a possibility that they have missed some features of connectivity to comprehensively test both hypotheses. Importantly, the abovementioned studies, which tested the directionality of connectivity, did not show opposite directions to the present work (i.e., opposite directionality would mean higher *in strength* in the interictal and higher *out strength* in the ictal period for non-SOZ than SOZ). In addition to the large set of connectivity measures and node metrics evaluated here, which assesses the connectivity more exhaustively, the larger sample size used here compared with those studies allows for a more powerful evaluation.

Our results suggest that rather than being the most central/connected in the network, the SOZ seems to separate from the rest of the network. It is important to note that our measures of connectivity were extracted from temporal patterns of activity. Therefore, the separation of the SOZ from the rest of the network is more in the temporal sense than spatial and may reflect a more complex and distributed network structure for the SOZ than the non-SOZ. In other words, while spatial proximity of areas can influence the similarity of their activities, separation here means dissimilarity in activity patterns rather than spatial location. This underlines the importance of considering temporal as well spatial features when investigating epileptogenic networks (Bartolomei et al., 2017). The separation of the SOZ is consistent with previous studies, which evaluated the temporal dynamics of network configurations. For example, it has been shown that, immediately after the seizure onset, the correlation in the whole-brain network drops significantly (Kerr et al., 2011; Schindler et al., 2007), possibly because of SOZ becoming functionally disconnected from other areas (Warren et al., 2010), which becomes less pronounced later in the seizure (indeed, hypercorrelation of EEG activity in the latter part of seizures has been postulated to be an emergent regulatory mechanism to promote seizure termination; Schindler et al., 2007). This is also probably why we observed generally higher discrimination of SOZ from non-SOZ immediately after the seizure compared with later epochs (cf., Supporting Information Figure S5). It is important to note that, while our results showed evidence for neural activity dominantly flowing towards the SOZ in the interictal periods, whether this neural activity is inhibitory remains unclear. This is because connectivity methods cannot determine whether the transmission is excitatory or inhibitory (Doss et al., 2024; Jiang et al., 2022; Lagarde & Bartolomei, 2024). LVFA, the hallmark of focal seizure onset across species, has been shown to be associated with increased firing in GABAergic inhibitory interneurons (Gentiletti et al., 2022), triggered by accumulation of extracellular potassium. Studies to further evaluate links between electrophysiologic seizure evolution and associated ionic and neurotransmitter changes (e.g., using optogenetic and pharmacological approaches in animal models) have helped advance understanding of the dynamics of focal seizures (Wenzel et al., 2023), but more investigation is needed and integrating directed connectivity methods into electrophysiologic models may be useful. Better understanding of the preictal to ictal transition may be of particular interest (Capitano et al., 2024).

Following more recent broadband data-driven approaches (Gunnarsdottir et al., 2022), we used broadband rather than narrowband signals in our analyses. This aligns with studies that evaluated the connectivity over the broad frequency bands and did not find any differences in directionality of signals across frequency bands (Doss et al., 2024; Jiang et al., 2022). Also, studies that suggested an effect of frequency on connectivity have reported inconsistent results. For example, while some studies have shown significantly higher *out/in degree* for EZ than non-EZ in the gamma band activity (Wang et al., 2017) and higher outward connectivity using single-pulse electrical stimulation (Johnson et al., 2023), other studies have reported a significant decrease in outgoing connectivity from the SOZ in the gamma band frequencies during seizures (Janca et al., 2021).

Previous studies have also shown that the information in the node metrics (i.e., connectomics), extracted from directed connectivity measures, could discriminate the SOZ from non-SOZ (Sethi et al., 2016; van Mierlo et al., 2013; Varotto et al., 2012; Vlachos et al., 2017; Wilke et al., 2011). Specifically, Wilke et al. (2011) found that the *betweenness centrality* was correlated with the location of resected cortical regions in patients with seizure-free outcomes. van Mierlo et al. (2013) found that the electrode contacts with the highest *out degree* always lay within the resected brain regions and that the patient-specific connectivity patterns were consistent over majority of seizures. Sethi et al. (2016) analysed a network constructed from fMRI data in patients with polymicrogyria and refractory epilepsy and found that the polymicrogyric nodes showed significantly increased *clustering coefficients* and *characteristic path lengths* compared with the normal contralateral homologous cortical regions. Varotto et al. (2012) analysed the connectivity pattern in patients with type II focal cortical dysplasia and found that *out density* can discriminate SOZ from non-SOZ. Vlachos et al. (2017) evaluated effective inflow obtained from several connectivity measures including (DCOH, PDCOH, and DTF) in the interictal period to show that EZ has a higher *inflow* than non-EZ. Higher *out degree* obtained from DTF in the ictal period accurately determined the EZ nodes in Yang et al. (2018). The present work is amongst the few that directly and quantitatively compared the information in several node metrics. Previously, Mao et al. (2016), who used PDC, have shown that *in degree* and *betweenness centrality* had more localisation information than *in degree* in the ictal period. In contrast, another study, which used nonlinear correlation, found more information in *out degree* than *in degree* (Courtens et al., 2016). The current study builds on these previous studies, combines a set of six node metrics extracted from 13 distinct connectivity measures to show how accurately they can discriminate SOZ from non-SOZ. We found evidence (BF > 10) for above-chance discrimination performance during both interictal and ictal windows, and the DI was amongst the most informative connectivity measures to localise the SOZ. We also found that *eccentricity* is even a more powerful biomarker for EZ localisation than *in strength* suggested in previous studies (Doss et al., 2024; Johnson et al., 2023).

It is of note that most of the previous studies have only indicated the discriminability of SOZ from non-SOZ contacts, rather than testing the generalisability of effects to new out-of-sample contacts. Our ML-based method learns the connectivity patterns from a set of contacts used for training and was able to discriminate the SOZ from non-SOZ in unseen/out-of-sample contacts (i.e., the contacts used to test the classifiers in the cross-validation process). The lower performance of our node metrics, compared with our recent multifeatural localisation method on the same dataset (Karimi-Rouzbahani & McGonigal, 2024), can be explained by a variety of reasons including a higher number of time windows incorporated in the analysis. It is of note that, while the results were above-chance, we did not optimise our classification/localisation pipeline; instead, we focused on showing the plausibility of the method for localisation. To make the algorithm ready for real-world application, further optimisations in the pipeline can be made from the machine learning literature such as incorporating univariate signal features (Karimi-Rouzbahani & McGonigal, 2024) and data augmentation. The optimisation of the proposed pipeline is the subject of future work.

Amongst the 13 connectivity measures tested in this study, only ANM supported both hypotheses. It may suggest that the patterns of activity and, in turn, connectivity significantly change from the interictal to the ictal period, which may lead to them being missed using any individual connectivity measure. More specifically, while the connectivity between brain areas might be facilitated through the modulation of signal complexities in the interictal period (as captured by CDS and DI; Figure 3A), the connectivity between brain areas might be facilitated by frequency-domain modulations in the ictal period (as captured by SGC; Figure 3B; Lagarde et al., 2019). This makes sense as ictal activities have been shown to strongly modulate the signal power in several frequency bands (Grinenko et al., 2018).

One of the limitations of our study was choosing a subsample of channels and mid-width windows for connectivity analysis, which were partially imposed by the computational load resulting from implementing a large set of connectivity measures and the sensitivity of some measures to the signal nonstationarity (Table 2). Future studies that desire to evaluate second-by-second temporal dynamics of connectivity using individual connectivity measures with less sensitivity to signal stationarity (e.g., PSI) can potentially use more channels and a higher number of time windows in analysis.

This work tested two critical hypotheses in epilepsy research and provided evidence that the SOZ seems to dominantly receive neural activities from non-SOZ potentially to be suppressed between seizures, whereas it dominantly transmits neural activities to non-SOZ during seizures. We showed that not all directed connectivity measures can detect those changes in connectivity direction from the interictal to ictal period, as probably the nature of connectivity changes with seizure onset. We also showed that, using a combination of node connectivity metrics extracted from directed connectivity measures, it is possible to localise the SOZ with above-chance performance. These results shed new light on the configuration of brain networks in epilepsy and introduces a potential method for localising the SOZ using explainable machine learning algorithms, as well as providing a rationalised set of measures for further investigation of seizure dynamics.

## ACKNOWLEDGMENTS

We thank the Mater Foundation and Mater Research Institute for supporting this study.

## SUPPORTING INFORMATION

Supporting information for this article is available at https://doi.org/10.1162/netn_a_00454.

## AUTHOR CONTRIBUTIONS

Hamid Karimi-Rouzbahani: Conceptualization; Data curation; Formal analysis; Investigation; Methodology; Software; Validation; Visualization; Writing – original draft; Writing – review & editing. Aileen McGonigal: Conceptualization; Funding acquisition; Supervision; Writing – review & editing.

## DATA AND CODE AVAILABILITY

The dataset used in this study was from previous studies and is available at https://openneuro.org/datasets/ds004100/versions/1.1.3. The readers are referred to the original studies for further details about the dataset including inclusion and exclusion criteria (Bernabei et al., 2022; Kini et al., 2019). The code developed for this project is available at https://github.com/HamidKarimi-Rouzbahani/Intracranial_epilepsy_connectivity.

## Note

^1^ In this work, we define the EZ as the areas primarily responsible for generating seizures (Lagarde & Bartolomei, 2024; Ryvlin et al., 2024), and the SOZ as where seizures start from (Bernabei et al., 2023). Nonetheless, this work analyses the directed connectivity only relative to the SOZ, but we try to use the term used in the original studies when reporting their results.

## Supplementary Material



## TECHNICAL TERMS

Focal epilepsy: One category of epilepsy where seizure activities start from a specific area of one brain hemisphere rather than both hemispheres.
Intracranial EEG: An electroencephalography method where the brain activity is recorded using electrodes inside the skull.
Epileptogenic zone (EZ): Brain areas primarily responsible for generating seizures in epilepsy.
Localisation: Discriminating brain areas where focal epileptic patterns start from other areas.
Seizure onset zone (SOZ): The area of the brain where epileptic seizures start from.
Connectivity measures: Methods that quantify relationship between activity patterns across brain areas.
Directed connectivity: A type of connectivity measured through directed connectivity measures that also consider the direction of activity flow between nodes.
Node metric: Measures that quantify how a node behaves in a network of interconnected nodes.
Sink SOZ: Hypothesis that SOZ areas dominantly receive brain activities rather than propagating them.
Source SOZ: Hypothesis that SOZ areas dominantly propagate brain activities rather than receiving them.
