# Supplementary material for: Directionality of neural activity in and out of the seizure onset zone in focal epilepsy
Source: Netw Neurosci. 2025 Jun 30;9(2):798–823. doi: 10.1162/netn_a_00454 (PMC12226147; doi:10.1162/netn_a_00454)
Supplement: Supplementary file 1 [file netn-9-2-798-s001.pdf]

Directionality of neural activity in and out of the seizure onset zone in focal epilepsy

Hamid Karimi-Rouzbahani,[1-3] Aileen McGonigal[1-3]

1 Neurosciences Centre, Mater Hospital, South Brisbane, 4101, Australia

2 Mater Research Institute, University of Queensland, South Brisbane, 4101, Australia

3 Queensland Brain Institute, University of Queensland, St Lucia, 4072, Australia

Correspondence to: Hamid Karimi-Rouzbahani

h.karimi-rouzbahani@uq.edu.au

Supplementary materials

Supplementary table 1 Demographics of patients.

Age	Sex	Hand	Outcome	Engel	Therapy	Implant	Resection target	Lesion status	Onset age
**40-49**	F	R	F	3A	ABLATION	SEEG	FRONTAL	n/a	10-19
**20-29**	M	L	S	1D	RESECTION	ECOG	FRONTAL	LESIONAL	0-9
**30-39**	M	R	S	1B	RESECTION	ECOG	TEMPORAL	LESIONAL	0-9
**30-39**	M	L	S	1B	RESECTION	ECOG	FRONTOPARIETAL	NON-LESIONAL	10-19
**20-29**	F	L	S	1C	RESECTION	ECOG	TEMPORAL	LESIONAL	0-9
**50-59**	F	L	F	4A	RESECTION	ECOG	TEMPORAL	NON-LESIONAL	50-59
**40-49**	F	L	F	2C	RESECTION	ECOG	TEMPORAL	NON-LESIONAL	30-39
**50-59**	F	R	S	1A	RESECTION	ECOG	TEMPORAL	LESIONAL	30-39
**20-29**	F	L	F	2A	RESECTION	ECOG	TEMPORAL	NON-LESIONAL	10-19
**20-29**	M	L	S	1D	RESECTION	ECOG	FRONTAL	LESIONAL	10-19
**30-39**	F	L	S	1D	RESECTION	ECOG	TEMPORAL	LESIONAL	0-9
**20-29**	M	n/a	S	1B	RESECTION	ECOG	TEMPORAL	LESIONAL	20-29
**40-49**	F	R	S	1B	RESECTION	ECOG	TEMPORAL	NON-LESIONAL	20-29
**30-39**	F	n/a	S	1D	RESECTION	ECOG	TEMPORAL	NON-LESIONAL	30-39
**30-39**	M	R	S	1A	RESECTION	ECOG	TEMPORAL	LESIONAL	20-29
**40-49**	F	L	S	1B	RESECTION	ECOG	TEMPORAL	NON-LESIONAL	20-29
**30-39**	M	R	S	1A	RESECTION	ECOG	TEMPORAL	NON-LESIONAL	0-9
**40-49**	F	R	S	1B	RESECTION	ECOG	TEMPORAL	NON-LESIONAL	20-29
**20-29**	F	R	F	3A	ABLATION	SEEG	FRONTAL	LESIONAL	0-9
**40-49**	F	n/a	F	3A	ABLATION	ECOG	MESIOTEMPORAL	NON-LESIONAL	20-29
**50-59**	F	R	S	1A	ABLATION	SEEG	MESIOTEMPORAL	LESIONAL	40-49
**30-39**	M	L	S	1A	RESECTION	SEEG	TEMPORAL	LESIONAL	10-19
**30-39**	M	n/a	S	1A	RESECTION	ECOG	TEMPORAL	LESIONAL	30-39
**20-29**	F	n/a	S	1A	ABLATION	ECOG	MESIOTEMPORAL	NON-LESIONAL	22
**40-49**	F	L	S	1B	ABLATION	SEEG	MFL	NON-LESIONAL	20-29
**50-59**	F	L	F	3A	ABLATION	SEEG	MESIOTEMPORAL	NON-LESIONAL	40-49
**30-39**	M	n/a	S	1B	RESECTION	SEEG	FRONTAL	LESIONAL	0-9
**30-39**	M	R	F	2A	ABLATION	SEEG	MESIOTEMPORAL	NON-LESIONAL	30-39
**30-39**	M	L	F	4A	ABLATION	SEEG	MESIOTEMPORAL	LESIONAL	20-29
**20-29**	M	L	S	1A	ABLATION	SEEG	PARIETAL	LESIONAL	0-9
**40-49**	F	L	S	1B	ABLATION	SEEG	MESIOTEMPORAL	NON-LESIONAL	20-29
**30-39**	M	R	S	1C	ABLATION	SEEG	MESIOTEMPORAL	NON-LESIONAL	10-19
**30-39**	M	n/a	S	1D	ABLATION	SEEG	MESIOTEMPORAL	LESIONAL	10-19
**30-39**	M	n/a	S	1D	RESECTION	SEEG	TEMPORAL	LESIONAL	0-9
**10-19**	M	n/a	S	1A	RESECTION	SEEG	TEMPORAL	NON-LESIONAL	0-9

**20-29**	M	n/a	S	1A	ABLATION	SEEG	TEMPORAL	LESIONAL	10-19
**10-19**	M	R	S	1B	ABLATION	SEEG	INSULAR	LESIONAL	0-9
**30-39**	M	R	F	2A	ABLATION	SEEG	MFL	NON-LESIONAL	0-9
**30-39**	M	R	F	3A	ABLATION	SEEG	INSULAR	NON-LESIONAL	0-9
**40-49**	F	n/a	S	1A	RESECTION	SEEG	TEMPORAL	NON-LESIONAL	10-19
**30-39**	F	n/a	F	3A	ABLATION	SEEG	MESIOTEMPORAL	NON-LESIONAL	10-19
**40-49**	F	L	S	1D	ABLATION	SEEG	MESIOTEMPORAL	NON-LESIONAL	10-19
**30-39**	F	L	S	1D	ABLATION	SEEG	MESIOTEMPORAL	LESIONAL	10-19
**20-29**	M	n/a	F	3A	RESECTION	SEEG	TEMPORAL	LESIONAL	0-9
**50-59**	M	L	F	2A	ABLATION	SEEG	FRONTAL	NON-LESIONAL	0-9
**20-29**	F	L	F	2A	ABLATION	SEEG	FRONTAL	NON-LESIONAL	0-9
**20-29**	F	R	S	1A	RESECTION	SEEG	TEMPORAL	LESIONAL	10-19
**40-49**	F	R	S	1A	RESECTION	SEEG	TEMPORAL	NON-LESIONAL	0-9
**20-29**	F	L	F	3A	RESECTION	SEEG	FRONTAL	LESIONAL	10-19
**20-29**	F	L	S	1A	ABLATION	SEEG	FRONTAL	LESIONAL	0-9
**30-39**	F	L	F	3A	ABLATION	SEEG	TEMPORAL	LESIONAL	10-19
**30-39**	M	L	S	1A	ABLATION	SEEG	MESIOTEMPORAL	LESIONAL	0-9
**20-29**	M	n/a	F	2A	ABLATION	SEEG	MESIOTEMPORAL	NON-LESIONAL	10-19
**20-29**	F	L	F	3A	RESECTION	SEEG	FRONTAL	LESIONAL	0-9
**20-29**	M	L	F	3A	RESECTION	SEEG	MESIOTEMPORAL	NON-LESIONAL	10-19

Supplementary text 1

Information theory measures

Additive Noise Model (ANM)

This measure assesses directed nonlinear dependence (or causality) of x → y under the assumption that the effect variable, y, is a function of a cause variable, x, along with an independent noise term (Hoyer et al., 2008). PySpi utilises the statistic from Causal Discovery Toolbox (CDT) as connectivity. This involves initially predicting y from x using a Gaussian process using a radial basis function kernel, followed by computing the normalized Hilbert-Schmidt Independence Criterion (HSIC) test statistic from the residuals. ANM is commonly used in causal inference studies, particularly when the underlying causal mechanisms are assumed to be deterministic and additive in nature.

Information-Geometric Causal Inference (IGCI)

This measure infers causal influence from x to y within deterministic systems featuring invertible functions (Janzing et al., 2012). In IGCI, causal inference is approached by examining the geometric structure of the joint probability distribution of variables. Specifically, IGCI focuses on estimating the causal influence of one variable (the cause) on another variable (the effect) by analysing the statistical dependencies between them. PySpi utilises CDT, where the difference in differential entropies is computed, with probability density estimated via nearest-neighbour estimators. One of the key features of IGCI is its ability to handle both linear and nonlinear causal relationships, making it applicable to a wide range of data types and systems. Additionally, IGCI can be used to infer causal relationships in scenarios where traditional statistical methods may not be suitable, such as when dealing with high-dimensional or noisy data.

Conditional Distribution Similarity Fit (CDS)

This measure provides a quantitative measure of the conditional relationship between variables and can help identify patterns of dependency or causality in empirical data (Cliff et al., 2023). It represents the standard deviation of the conditional probability distribution of y given x. This involves estimating the conditional probability distributions by discretising the values of the x and y and then computing the standard deviation of these conditional distributions. CDSF does not rely on specific parametric models to describe the relationship between variables, which makes it ideal for objective analyses.

Regression Error-Based Causal Inference (RECI)

This provides an assessment of the causal impact of x → y by measuring the error in a regression of y on x using a monomial (power product) model (Blöbaum et al., 2018). The rationale behind this method is that if there is a causal relationship from x to y, the regression model should capture most of the variation in y. This statistic corresponds to the Mean Squared Error (MSE) resulting from the linear regression of the cubic (with a constant term) of x with y. While linear regression models are commonly used, RECI can also be extended to handle nonlinear relationships between variables.

Causally Conditioned Entropy (CCE)

This measure quantifies the remaining uncertainty in time series y given the entire causal past of both time series x and y (Cliff et al., 2023). It is computed as a sum of conditional entropies of y given the past of both x and y with increasing history lengths. CCE is a sophisticated measure for assessing the causal influence of one time series on another by quantifying the remaining uncertainty in the target series after conditioning on the past values of both series. It is a versatile tool that can handle both linear and nonlinear dependencies. For computational efficiency, PySpi sets the history length of 10. This implies that the joint process is assumed to be, at most, a 10th-order Markov chain. We used a Gaussian kernel in this work.

Directed Information (DI)

It is a measure for assessing the information flow from a source time series x to a target time series y (Massey, 1990). It is calculated as the difference between the conditional entropy of y given its own past and the *CCE*. This measure provides an interpretable framework for understanding causal influence, as it directly quantifies the amount of information transferred from the source to the target time series. As in *CCE*, the computation of *directed information* is limited to a history length of 10. We used a Gaussian kernel in this work.

Frequency-domain measures

Group Delay (GD)

Group delay quantifies a directed, average time delay between two signals by assessing the slope of the phase differences as a function of frequency (derived through linear regression) (Hannan & Thomson, 1973). This slope is computed solely for coherence values that are statistically significant, and the time delay is acquired through a straightforward rescaling of the slope by 2π. The implementation provides the output in the form of the rescaled time delay statistic. GD's ability to

provide directional, frequency-dependent, and time-resolved measures of the interactions between signals is valuable. Its reliance on phase differences makes it particularly effective for studying the temporal dynamics of complex systems.

Phase Slope Index (PSI)

This measure serves as a directed metric for assessing information flow, computed using the complex-valued coherence (Nolte et al., 2008). Specifically, it evaluates the consistency of phase difference alterations across a predefined frequency range, with coherence acting as a weighting factor. The implementation computes the measure in the frequency domain. Its reliance on phase coherence makes it robust to noise and effective in identifying directed connectivity within specific frequency bands. However, *PSI* primarily assumes linear relationships in the phase domain. Nonlinear interactions may not be fully captured by this measure. Moreover, accurate phase difference estimation requires high-quality signals. Pre-processing steps such as filtering and artefact removal are crucial for reliable *PSI* computation.

Directed Transfer Function (DTF)

This measure uses cross-spectral density matrix which can be decomposed into a noise covariance matrix and a spectral transfer matrix (Eichler, 2006). The *directed transfer function* is derived from this decomposition to quantify the inflow from x to y. This inflow is normalized by the total inflow from all other signals into y, represented by the row-wise sum of the spectral transfer matrix. It can provide frequency-specific insights into connectivity, and its normalisation allows for direct comparison of connectivity between distinct pairs of signals. However, the computationally intensive nature of the method, its assumption linearity limits the application of DTF in detecting nonlinear relationships.

Directed Coherence (DCOH)

It is calculated from the inflow from x to y using the spectral transfer matrix (as described in DTF) and is then normalised by their noise covariance (Baccalá & Sameshima, 2001). It can provide frequency-specific insights into connectivity, and its normalisation allows for direct comparison of connectivity between distinct pairs of signals. However, the computationally intensive nature of the method, its assumption linearity limits the application of DCOH in detecting nonlinear relationships. Accurate estimation of *DCOH* requires high-quality signals. Noise and artefacts in the data can affect the reliability of the results, necessitating careful pre-processing steps.

Partial Directed Coherence (PDCOH)

The partial directed coherence from x to y is determined by the inflow (as described in DTF), normalized by the total outflow from all other signals into y (the column-wise sum of the spectral transfer matrix) (Baccalá & Sameshima, 2001). As an advantage to *DCOH*, by considering the influence of all other signals in the network (on the signals being evaluated), *PDCOH* provides a more accurate assessment of the true directional relationships between specific signal pairs.

Spectral Granger Causality (SGC)

This measure extends the concept of Granger Causality to the frequency domain, enabling the assessment of causal interactions between signals at specific frequencies (Friston et al., 2014). It is calculated using the spectral transfer matrix and noise covariance. These are estimated through either a parametric (VAR model) approach or a nonparametric (spectral factorization) approach. We used the *nonparametric* method to minimise subjective parameter settings. In the PySpi toolbox is implemented using the Spectral Connectivity Toolbox. *SGC's* ability to provide frequency-specific and normalised measures of causality makes it particularly useful where understanding the dynamics of complex systems is important. However, its reliance on linearity and sensitivity to signal quality should be considered when interpreting the results.

Time-domain measure

Linear Model Fit (LMFIT)

Linear regression is a widely employed method for assessing independence via model fittings (Cliff et al., 2023). We employed ridge regression from the toolbox, which uses $\ell 2$-norm regularization and the mean squared error (MSE) resulting from a regression of y on x. This measure is a powerful and widely used statistical method for modelling the directed relationship between signals. Its simplicity, interpretability, and efficiency make it a valuable tool for relationship analysis, and trend analysis in various fields of study. However, it has several limiting assumptions including that observations are independent of each other. Violation of this assumption (e.g., autocorrelation in time series data) can lead to inaccurate estimates and predictions. Linear regression assumes a linear relationship between signals. If the true relationship is nonlinear, the model may provide biased or inaccurate results. Finally, overfitting can occur when the model is too complex relative to the amount of available data. This can result in poor generalization to new data and unreliable predictions.

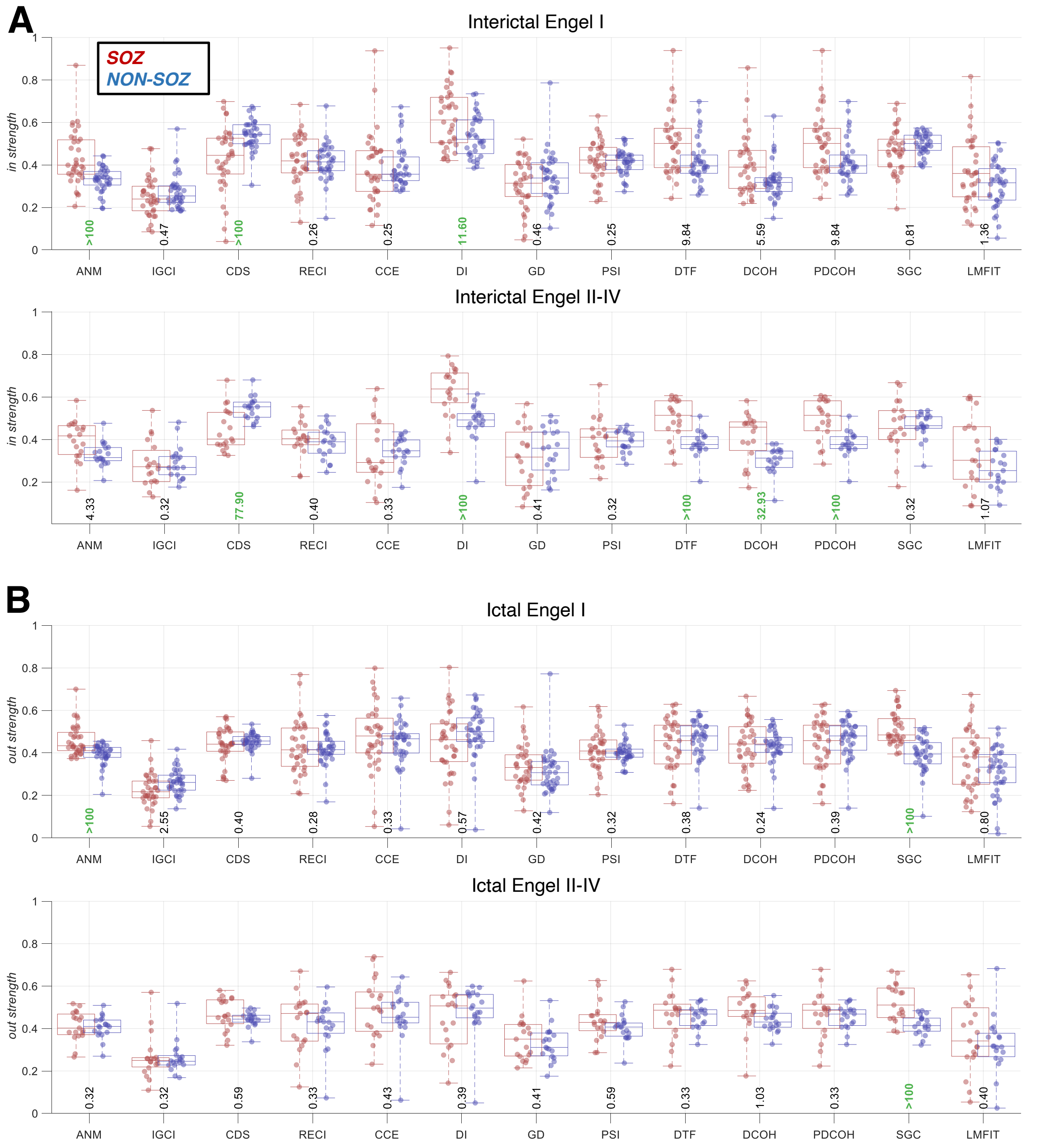

Supplementary Figure 1 *In strength* in the interictal (A) and *out strength* in the ictal (B) period across connectivity measures separated for seizure-free and non-seizure-free patients. Results are separated for the SOZ (red) and non-SOZ (blue) contacts with each dot showing data from one patient. Box plots show the distribution of data, its quartiles and median and whiskers indicate the maximum and minimum of the data over patients. Numbers below the bars indicate Bayesian evidence (BF > 10 indicated in green) for the difference between SOZ and non-SOZ data.

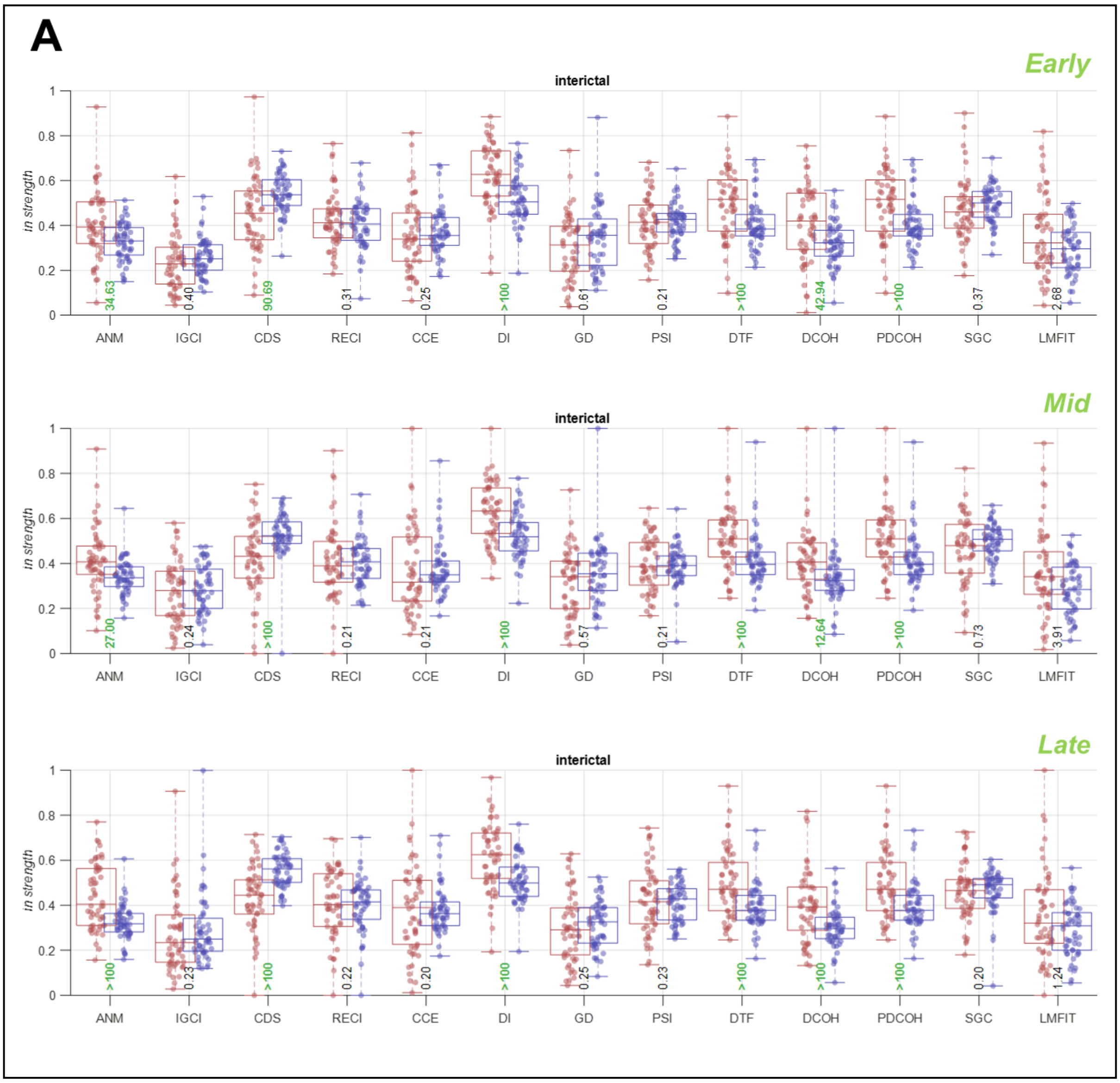
A
Early
interictal
in strength
ANM IGCI CDS RECI CCE DI GD PSI DTF DCOH PDCOH SGC LMFIT
34.63 0.40 90.69 0.31 0.25 >100 0.61 0.21 >100 42.94 >100 0.37 2.68
Mid
interictal
in strength
ANM IGCI CDS RECI CCE DI GD PSI DTF DCOH PDCOH SGC LMFIT
27.00 0.24 >100 0.21 0.21 >100 0.57 0.21 >100 12.64 >100 0.73 3.91
Late
interictal
in strength
ANM IGCI CDS RECI CCE DI GD PSI DTF DCOH PDCOH SGC LMFIT
>100 0.23 >100 0.22 0.20 >100 0.25 0.23 >100 >100 >100 0.20 1.24

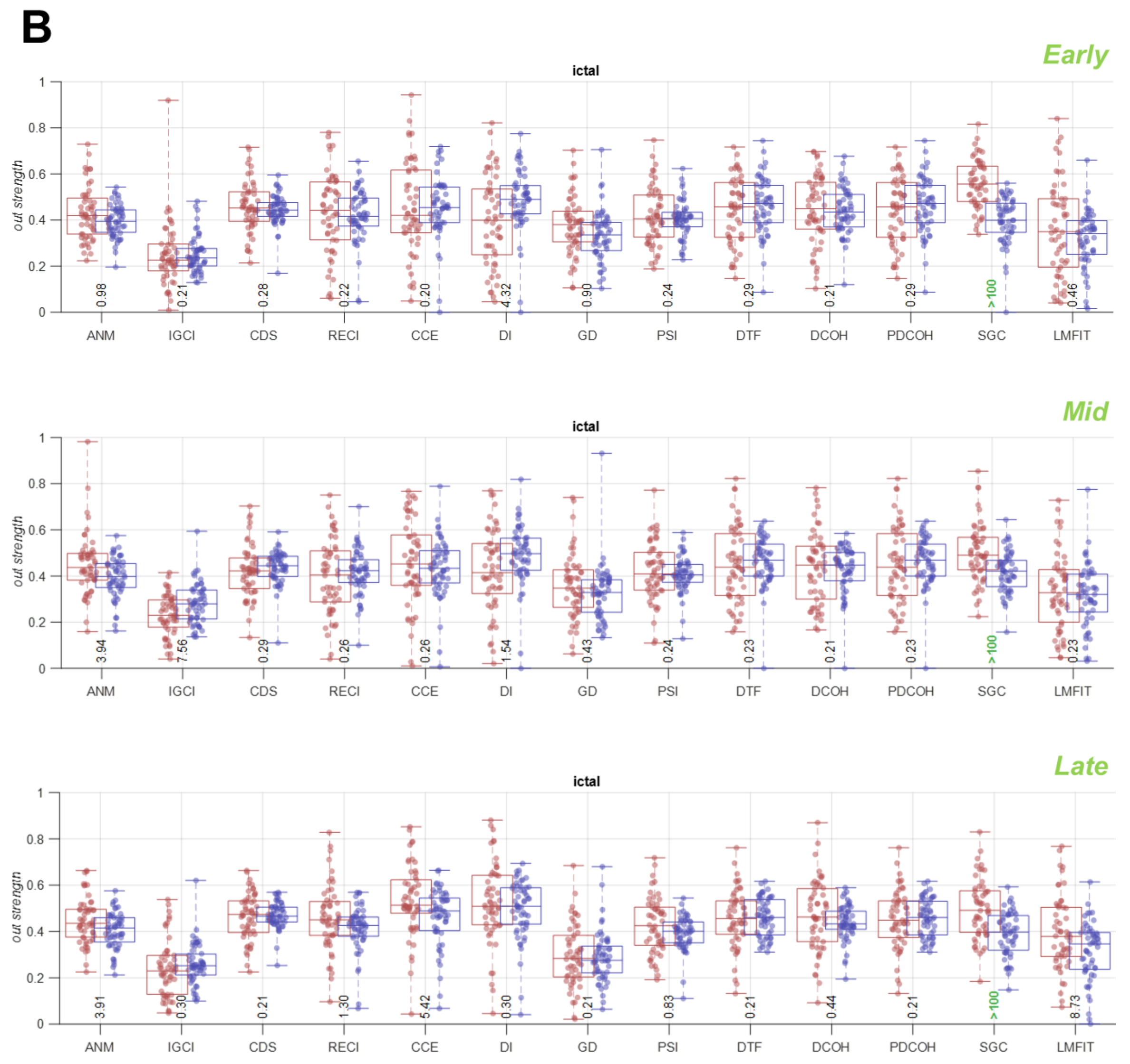

Supplementary Figure 2 *In strength* in the interictal (A) and *out strength* in the ictal (B) period across connectivity measures using different epochs of data. Results are separated for the SOZ (red) and non-SOZ (blue) contacts with each dot showing data from one patient. Box plots show the distribution of data, its quartiles and median and whiskers indicate the maximum and minimum of the data over patients. Numbers below the bars indicate Bayesian evidence (BF > 10 indicated in green) for the difference between SOZ and non-SOZ data.

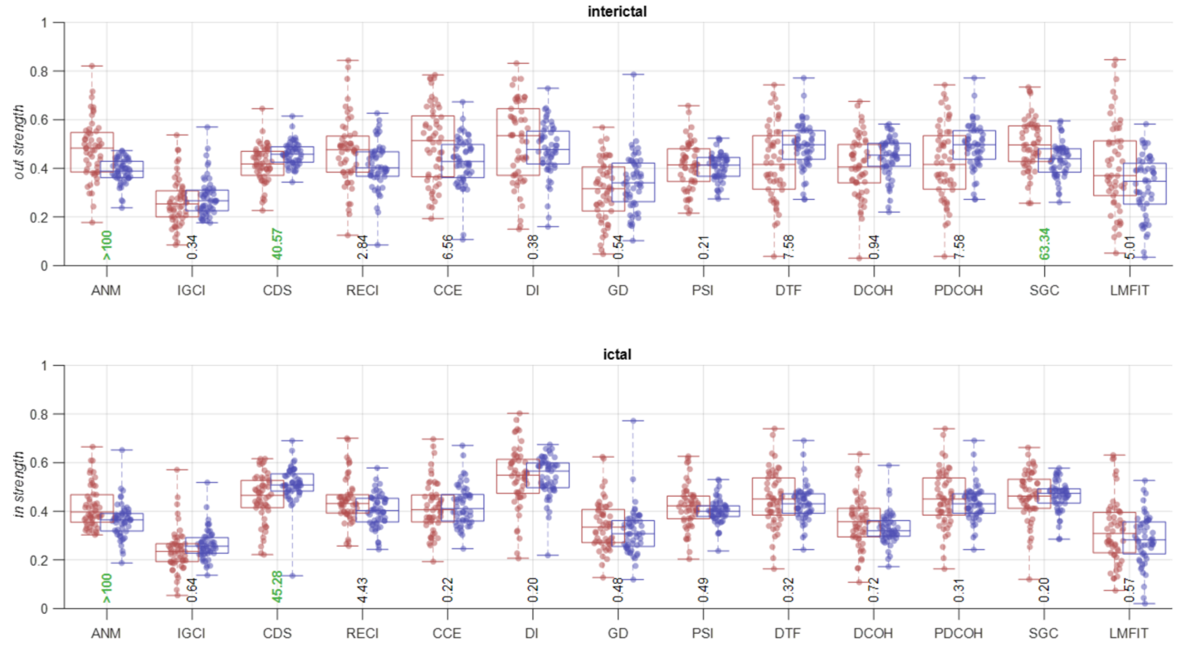

Supplementary Figure 3 ***Out strength*** **in the interictal (top) and** ***in strength*** **in the ictal (bottom) period across connectivity measures.** Results are separated for the SOZ (red) and non-SOZ (blue) contacts with each dot showing one patient. Box plots show the distribution of data, its quartiles and median and whiskers indicate the maximum and minimum of the data over patients. Numbers below the bars indicate Bayesian evidence (BF > 10 indicated in green) for the difference between SOZ and non-SOZ data.

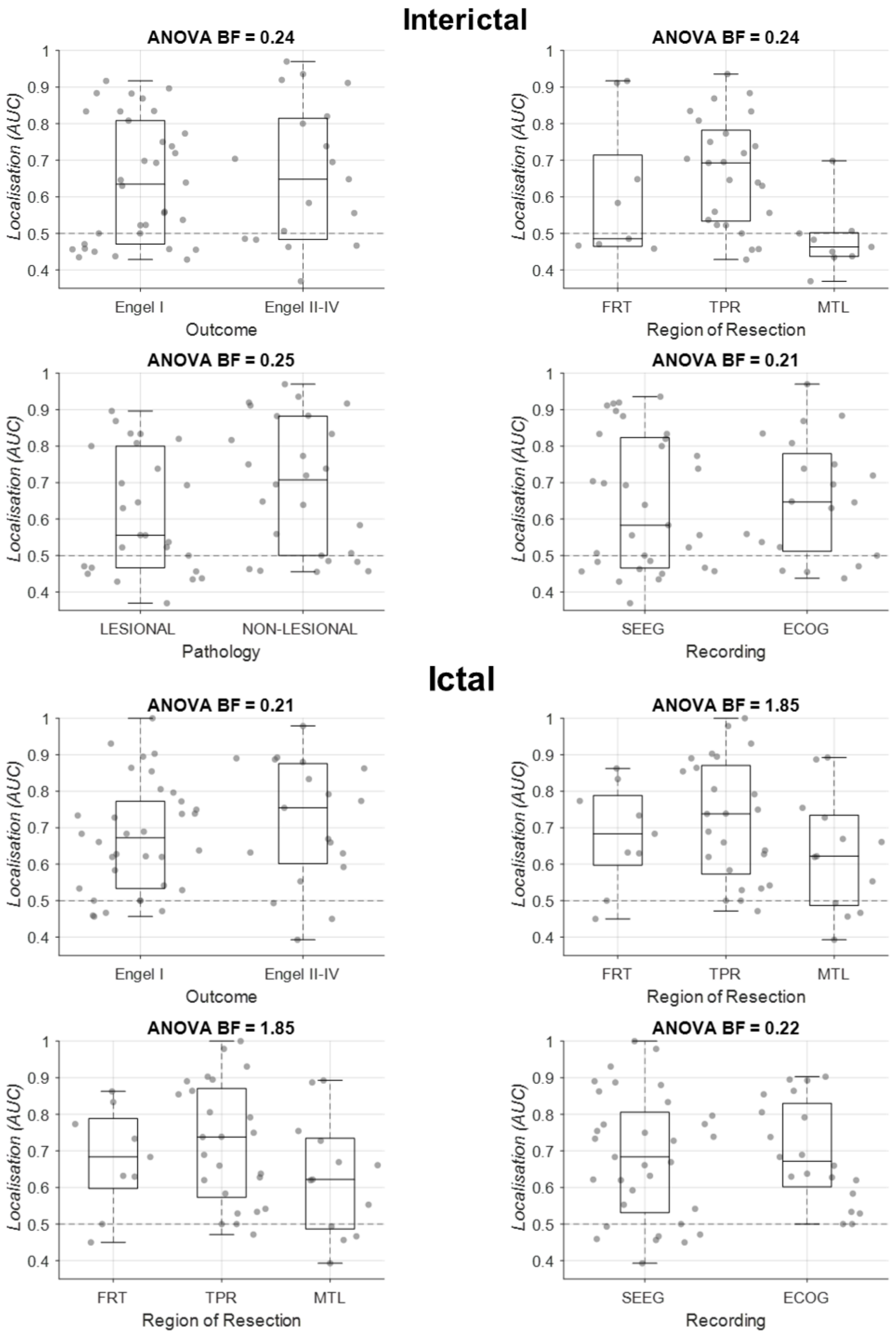

Supplementary Figure 4 Main effects of surgery outcome (Engel I/Engel II-IV), region of resection (FRT/TPR/MTL), pathology (lesional/non-lesional), and recording modality (SEEG/ECoG) on SOZ localisation. Effects were calculated using Bayes factor ANOVA. Top and bottom panels show the results for interictal and ictal classification analysis, respectively. Box plots show the distribution of data, its quartiles and median and whiskers indicate the maximum and minimum of the data across patients. Each dot indicates the data from one patient. Horizontal dashed line refers to theoretical chance-level classification (0.5). The number on the top of each panel indicates Bayesian evidence for an effect on classification performance. FRT: frontal; TPR: temporal; MTL: mesiotemporal.

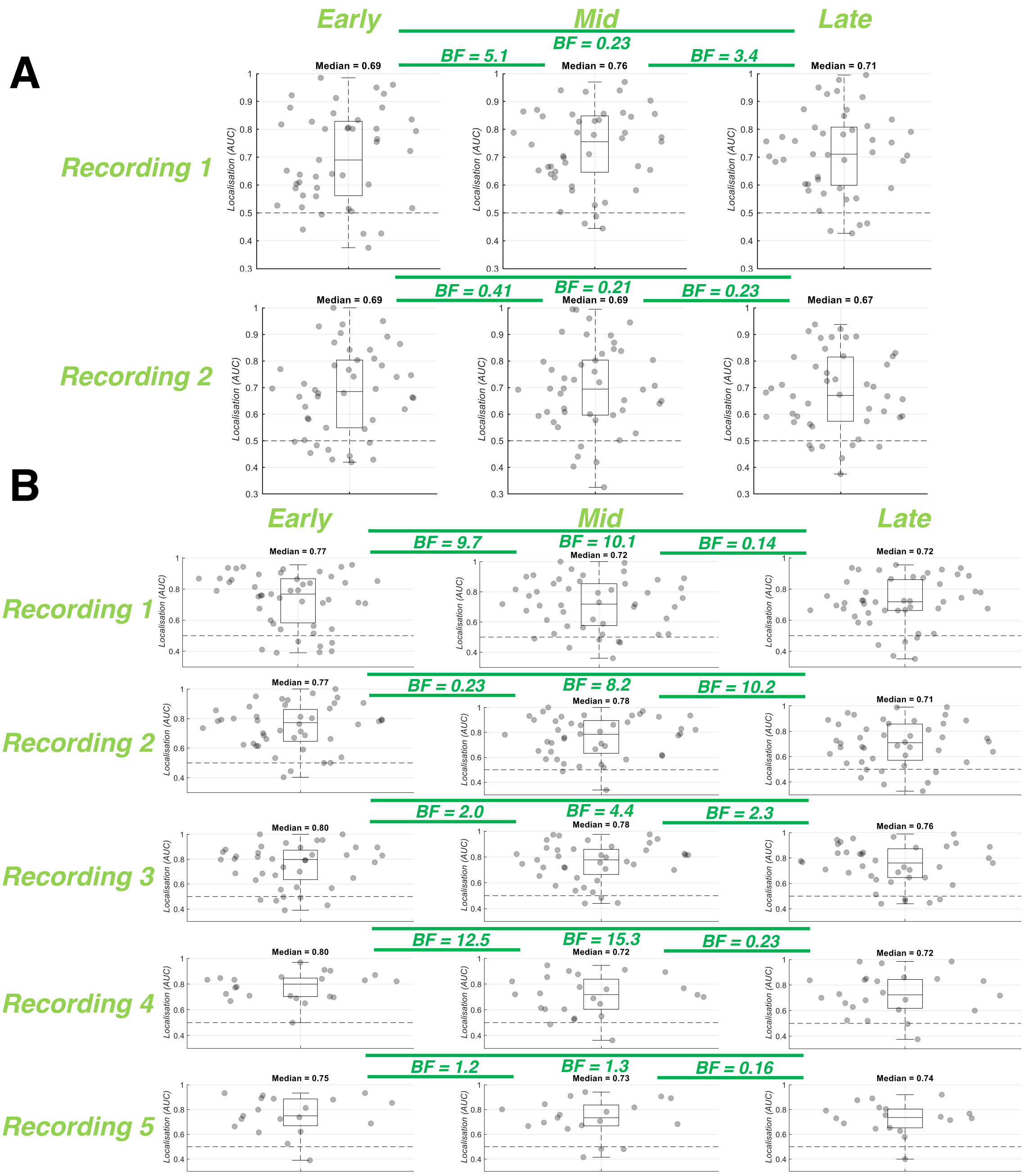

Supplementary Figure 5 Classification of SOZ and non-SOZ contacts in different epochs of data. AUC of classification performance for interictal **(A)** and ictal **(B)** data in different recordings and epochs. Each patient had 2 interictal recordings and 1 to 5 ictal recordings. Box plots show the distribution of data, its quartiles and median and whiskers indicate the maximum and minimum of the data over patients. Each dot indicates the data from one patient. All classification results were above chance (BF > 10; Bayesian evidence for the difference between true and null generalisation performances). Horizontal dashed line refers to theoretical chance-level classification (0.5). Horizontal solid lines show which epochs are compared.

References

Baccalá, L. A., & Sameshima, K. (2001). Partial directed coherence: a new concept in neural structure determination. *Biological Cybernetics*, *84*(6), 463–474. https://doi.org/10.1007/PL00007990

Blöbaum, P., Janzing, D., Washio, T., Shimizu, S., & Schölkopf, B. (2018). *Cause-Effect Inference by Comparing Regression Errors*.

Cliff, O. M., Bryant, A. G., Lizier, J. T., Tsuchiya, N., & Fulcher, B. D. (2023). Unifying pairwise interactions in complex dynamics. *Nature Computational Science*, *3*(10), 883–893. https://doi.org/10.1038/s43588-023-00519-x

Eichler, M. (2006). On the evaluation of information flow in multivariate systems by the directed transfer function. *Biological Cybernetics*, *94*(6), 469–482. https://doi.org/10.1007/s00422-006-0062-z

Friston, K. J., Bastos, A. M., Oswal, A., van Wijk, B., Richter, C., & Litvak, V. (2014). Granger causality revisited. *NeuroImage*, *101*, 796–808. https://doi.org/10.1016/j.neuroimage.2014.06.062

HANNAN, E. J., & THOMSON, P. J. (1973). Estimating group delay. *Biometrika*, *60*(2), 241–253. https://doi.org/10.1093/biomet/60.2.241

Hoyer, P. O., Janzing, D., Mooij, J., Peters, J., & Schölkopf, B. (n.d.). *Nonlinear causal discovery with additive noise models*.

Janzing, D., Mooij, J., Zhang, K., Lemeire, J., Zscheischler, J., Daniušis, P., Steudel, B., & Schölkopf, B. (2012). Information-geometric approach to inferring causal directions. *Artificial Intelligence*, *182–183*, 1–31. https://doi.org/10.1016/j.artint.2012.01.002

Massey, J. L. (1990). CAUSALITY, FEEDBACK AND DIRECTED INFORMATION. In *Intl. Symp. on Info. Th. and its Applications*.

Nolte GUIDONOLTE, G., Ziehe ZIEHE, A., Krämer NKRAEMER, N., Popescu FLORINPOPESCU, F., Nolte, G., Ziehe, A., Krämer, N., Popescu, F., & Müller NOLTE ZIEHE KRÄMER POPESCU MÜLLER, K. (n.d.). Comparison of Granger Causality and Phase Slope Index Klaus-Robert Müller. In *JMLR Workshop and Conference Proceedings* (Vol. 6).
